# Family Conferences to Facilitate Deprescribing in Older Outpatients With Frailty and With Polypharmacy: The COFRAIL Cluster Randomized Trial

**DOI:** 10.1001/jamanetworkopen.2023.4723

**Published:** 2023-03-27

**Authors:** Achim Mortsiefer, Susanne Löscher, Yekaterina Pashutina, Sara Santos, Attila Altiner, Eva Drewelow, Manuela Ritzke, Anja Wollny, Petra Thürmann, Veronika Bencheva, Matthias Gogolin, Gabriele Meyer, Jens Abraham, Steffen Fleischer, Andrea Icks, Joseph Montalbo, Birgitt Wiese, Stefan Wilm, Gregor Feldmeier

**Affiliations:** 1 Institute of General Practice and Primary Care, Chair of General Practice II and Patient-Centredness in Primary Care, Faculty of Health, Witten/Herdecke University, Witten, Germany; 2 Institute of General Practice, Medical Faculty, Heinrich-Heine-University Düsseldorf, Düsseldorf, Germany; 3 Institute of General Practice, University Medical Center Rostock, Rostock, Germany; 4 Department of Clinical Pharmacology, School of Medicine, Faculty of Health, Witten/Herdecke University, Witten, Germany; 5 Institute for Health and Nursing Science, Medical Faculty, Martin Luther University Halle-Wittenberg, Halle (Saale), Germany; 6 Institute for Health Services and Economics, Centre for Health and Society, Faculty of Medicine, Heinrich-Heine-University Düsseldorf, Düsseldorf, Germany; 7 WG Medical Statistics and IT-Infrastructure, Institute of General Practice, Hannover Medical School, Hannover, Germany

## Abstract

**Importance:**

For older adults with frailty syndrome, reducing polypharmacy may have utility as a safety-promoting treatment option.

**Objective:**

To investigate the effects of family conferences on medication and clinical outcomes in community-dwelling older adults with frailty receiving polypharmacy.

**Design, Setting, and Participants:**

This cluster randomized clinical trial was conducted from April 30, 2019, to June 30, 221, at 110 primary care practices in Germany. The study included community-dwelling adults aged 70 years or older with frailty syndrome, daily use of at least 5 different medications, a life expectancy of at least 6 months, and no moderate or severe dementia.

**Interventions:**

General practitioners (GPs) in the intervention group received 3 training sessions on family conferences, a deprescribing guideline, and a toolkit with relevant nonpharmacologic interventions. Three GP-led family conferences for shared decision-making involving the participants and family caregivers and/or nursing services were subsequently held per patient at home over a period of 9 months. Patients in the control group received care as usual.

**Main Outcomes and Measures:**

The primary outcome was the number of hospitalizations within 12 months, as assessed by nurses during home visits or telephone interviews. Secondary outcomes included the number of medications, the number of European Union list of the number of potentially inappropriate medication (EU[7]-PIM) for older people, and geriatric assessment parameters. Both per-protocol and intention-to-treat analyses were conducted.

**Results:**

The baseline assessment included 521 individuals (356 women [68.3%]; mean [SD] age, 83.5 [6.17] years). The intention-to-treat analysis with 510 patients showed no significant difference in the adjusted mean (SD) number of hospitalizations between the intervention group (0.98 [1.72]) and the control group (0.99 [1.53]). In the per-protocol analysis including 385 individuals, the mean (SD) number of medications decreased from 8.98 (3.56) to 8.11 (3.21) at 6 months and to 8.49 (3.63) at 12 months in the intervention group and from 9.24 (3.44) to 9.32 (3.59) at 6 months and to 9.16 (3.42) at 12 months in the control group, with a statistically significant difference at 6 months in the mixed-effect Poisson regression model (*P* = .001). After 6 months, the mean (SD) number of EU(7)-PIMs was significantly lower in the intervention group (1.30 [1.05]) than in the control group (1.71 [1.25]; *P* = .04). There was no significant difference in the mean number of EU(7)-PIMs after 12 months.

**Conclusions and Relevance:**

In this cluster randomized clinical trial with older adults taking 5 or more medications, the intervention consisting of GP-led family conferences did not achieve sustainable effects in reducing the number of hospitalizations or the number of medications and EU(7)-PIMs after 12 months.

**Trial Registration:**

German Clinical Trials Register: DRKS00015055

## Key Points

**Question:**

Do general practitioner–led family conferences promoting deprescribing in older adults with frailty and polypharmacy result in fewer hospitalizations?

**Findings:**

In this cluster randomized trial of 521 community-dwelling older adults with frailty and polypharmacy, the number of hospitalizations over 12 months did not differ significantly among those who received a maximum of 3 family conferences. The number of potentially inappropriate medications decreased significantly in the intervention group after 6 months, but the reduction was not retained at 12 months.

**Meaning:**

The findings of this trial suggest that family conferences for shared decision-making can successfully initiate the process of discontinuing medication, but no clinical benefit in terms of hospitalization was found.

## Teaser

This cluster randomized trial evaluates the effects of general practitioner–led family conferences on deprescribing and hospitalizations in community-dwelling older adults with frailty.

## Introduction

In older adults with frailty,^1^ polypharmacy—usually described as the use of 5 drugs or more^2^—is considered a major risk factor for poor health outcomes such as falls, delirium, malnutrition, hospitalization, and mortality.^3,4^ Adverse drug reactions are found in 35% of older community-dwelling adults and general drug-related problems are responsible for 10% to 30% of hospitalizations in these patients.^5,6,7^ The safety of drug therapy in older adults with frailty is affected not only by adherence, the number of drugs used, and prescribing cascades,^8^ but also by the use of European Union number of potentially inappropriate medication (EU[7]-PIM) for older people.^9,10^ Some studies have been able to improve the quality of drug prescriptions and reduce the number of EU(7)-PIMs taken,^11,12^ while others have not achieved this goal.^13,14,15^ However, it is unclear whether these interventions to reduce inappropriate prescribing led to clinically relevant improvements, so the need for further studies on clinical end points has been raised.^16,17,18^ A relevant general clinical end point that has been investigated in several studies and has yielded inconsistent results is the number of hospitalizations. A Cochrane review by Rankin et al^19^ reported that hospitalizations were reduced in 4 of 12 studies included, but no significant change was seen in the other 8 studies. However, the number of hospitalizations, compared with other broad clinical parameters, is an outcome that is relevant to both patients and the health system and should therefore be explored in more detail in further research.^20^

Most of the recent pragmatic (ie, conducted under everyday conditions) interventions to facilitate the process of prioritizing and discontinuing medications for older patients with polypharmacy have relied on electronic decision support^21,22^ or pharmacist advice.^23^ However, many barriers to reducing inappropriate polypharmacy have been identified among physicians, patients, and relatives, leading to major challenges in communication between the stakeholders involved.^24,25^ The process of deprescribing is complex and should therefore be promoted not only through better information for health care professionals but also through improved communication between all stakeholders.^26,27^ For older adults living at home, family caregivers often play a central role in medication management.^28^ Therefore, interventions to reduce polypharmacy should be embedded in a shared decision-making process between general practitioners (GPs), patients, and family caregivers.^29^

Family conferences are a well-established instrument in nursing and medical care (eg, in intensive care units or palliative care).^30,31,32^ Although family conferences help improve the communication process^30^ and patients with frailty can be included in conference planning,^32^ to our knowledge, no interventional study has investigated their impact on drug safety in older adults with frailty cared for at home.

The aim of this study was to investigate the effects of GP-led family conferences for shared decision-making on deprescribing in community-dwelling older adults with frailty who were receiving polypharmacy. The main hypothesis was that patient safety, reflected as a decrease in hospital admissions, would improve. This intended effect of the COFRAIL intervention, which comprises education and family conferences, should be achieved, on the one hand, directly by reducing adverse drug reactions and, on the other hand, indirectly through better medication adherence and more attentive and individually tailored treatment. The secondary hypothesis related to the decrease in the number and inappropriateness of medications in terms of EU(7)-PIMs.^33^

## Methods

### Study Design

We conducted a pragmatic cluster randomized clinical trial to evaluate the COFRAIL intervention in comparison with usual care in Germany under everyday conditions.^34^ The study protocol was published elsewhere^33^ and the submitted and approved version is available in Supplement 1. This study was approved by the ethics committees of the study centers in Rostock and Düsseldorf and was supervised by a data safety monitoring board. Eligible participants were approached by their GP, received an information letter with data protection details, and were asked to sign a consent form; participating GPs also signed an informed consent form. The GPs received financial compensation. The trial followed the Consolidated Standards of Reporting Trials (CONSORT) reporting guideline with extension for cluster trials. The study period was April 30, 2019, to June 30, 2021.

### Recruitment and Randomization

A random selection of GPs was gradually contacted during the study period by the study centers by post or email followed by telephone calls or practice visits to provide information and conduct recruitment. Once recruited, the GPs were asked to consecutively approach potentially suitable study participants from their list of older patients and enroll them in the study until an average number of 5 participants per practice (cluster) was reached. The GPs were not aware of their group assignment at the time of patient recruitment. Cluster randomization was conducted at the practice level, in which all participating GPs from a practice were assigned together to either the intervention or control group. The allocation sequence was computer generated and concealed from researchers. Block randomization (block size 4) and stratification by centers were used to ensure equal distribution across the study sites. An experienced biometrician at Hannover Medical School (B.W.) performed randomization after recruitment of the participants.

### Participants

Participant inclusion factors were a positive GP rating for frailty syndrome at levels 5 to 7 of the extended Clinical Frailty Scale, Version 1.2,^35^ aged 70 years or older, and regular intake of at least 5 different drugs per day. In addition, participants must already have a need for care in the activities of daily living and/or the instrumental activities of daily living confirmed by a previous routine assessment by the German long-term care insurance system; the care can be provided by family members or a professional caregiver. Exclusion criteria were moderate or severe dementia, a life expectancy of 6 months or less, living in a nursing home, insufficient German language skills of the patient and family caregivers, or nonavailability of an interpreter.

### Intervention

The COFRAIL intervention consisted of 2 steps. The 2 steps consisted of 3 education sessions and 3 family conferences.

#### Step 1: Education

Participating GPs were invited to 3 consecutive face-to-face educational sessions over a period of 6 months, of which 2 were mandatory and 1 was optional (conducted mainly in an online format as of April 2020 due to the COVID-19 pandemic). The workshops included case-based teaching of the contents of a deprescribing guideline and a geriatric toolbox on nonpharmacologic interventions, as well as communication training on how to conduct structured family conferences.

#### Step 2: Family Conferences

The GPs were asked to conduct a total of 3 family conferences per patient at home, 1 each at the beginning of the study (after completion of baseline data collection by study nurses) and at 3 and 9 months, each lasting 30 to 45 minutes, with the involvement of family caregivers and/or the ambulatory care service. In the joint discussion, GPs were asked to (1) determine the general preferences of the patient, (2) perform a medication check, (3) address further topics from the nonpharmacologic toolbox as needed, and (4) make follow-up appointments. The medication check was to include a review of patient-acquired medication from a pharmacy and, for each medication, an assessment of whether the medication was (still) necessary, whether the risks outweigh the potential benefits, and whether the prescription was consistent with the patient's current treatment preferences.^36^ The rationale and development of the COFRAIL intervention, including a deprescribing manual, has been published elsewhere.^36,37^

### Control

The participants of the control group received care as usual. The GPs in the control group were offered voluntary training events on geriatric topics and were invited to attend a training session on the contents of the COFRAIL intervention after completion of data collection.

### Outcome Measures

The observation period per participant was 12 months with data collection at T0 (baseline), T1 (6-month follow-up), and T2 (12-month follow-up). Data collection was performed by study nurses through participant interviews during home visits. With the onset of the COVID-19 pandemic, the study nurses began using telephone interviews in mid-March 2020. The proportion of telephone data collection by the study nurses was 26.5% at baseline, 66.6% at T1, and 93.4% at T2. Additional data on medical diagnoses, hospitalizations in the past 6 months, and the safety parameters (blood pressure, fasting glucose serum level, and glomerular filtration rate) were collected by the GPs based on patient records. At the time of the baseline survey, study nurses and GPs were blinded.

The primary outcome of the study was defined as the mean number of hospitalizations (with at least 1 overnight stay) per patient within 12 months after the start of the study. In each of the 3 data collections, the number of hospital admissions in the past 6 months was recorded. The maximum of the patient's indication (collected by the study nurse) and the GP’s indication (collected by a questionnaire) was regarded as valid information for hospitalizations. In the case of dropout due to death or other reasons, the primary outcome was collected by the GP.

Secondary pharmacologic outcomes were the total number of medications taken in the past 7 days and the EU(7)-PIMs for older people.^10^ All medications taken regularly by participants (including over-the-counter medications or vitamins/supplements) were recorded by the study nurses during home visits or telephone calls. In addition, a geriatric assessment was carried out by the study nurses (eTable in Supplement 2).

### Statistical Analysis

With an observation period of 12 months, 0.75 hospitalizations per patient were assumed, derived from a comparable population (AgeCoDe study).^38^ We consider the prevention of a mean of 0.25 hospitalizations per patient as sufficiently clinically relevant to justify implementation of the time-consuming and costly COFRAIL intervention in routine care. If the intervention is to reduce the number of hospitalizations by 33% from 0.75 to 0.5, 253 participants per group would be required (assuming an SD of 1 stay) to demonstrate this effect (α error, .05, power 80%, 2-sided *t* test). Assuming an intracluster correlation of 0.05 and a mean of 5 participants per cluster, the design effect is 1.2. Thus, 608 participants in 112 practices would be required. Assuming a dropout rate of 10% among participants and physicians, a total of 676 participants in 136 practices had to be recruited.

Descriptive statistical methods and multilevel (mixed) regression models were used for data analysis. Depending on the distribution of the outcome variable, Poisson regression, linear regression, or logistic regression models were applied. To take into account the cluster effect, GP practice identification was included in the model as a random effect. For the primary outcome, an intention-to-treat (ITT) analysis was performed, including all participants recruited and participating at baseline and with information about the hospitalization status at the end of the observation period. Missing values were not imputed.

The adjusted number of hospitalizations was calculated by dividing the number of hospitalizations by the actual number of observation days and then multiplying it by 365.25 days. The multivariate analyses were performed using the unadjusted number of hospitalizations; for the adjustment, the actual observation time was included as covariate. Other covariates used for adjustment were age in years (integer), sex (nominal), number of chronic diseases (integer), and number of hospitalizations (integer) in the 6 months before baseline. A sensitivity analysis was performed with the per-protocol (PP) population consisting of all participants participating in the T2 visit and participants in the intervention group who had at least 2 family conferences.

Data on the secondary outcomes were not collected for participants from whom no data were collected at T2 (dropouts), which means that the ITT analyses for the secondary outcomes would more or less consist of the participants in the PP population due to the number of missing values. For the PP analyses, only a few participants who did not complete the intervention were excluded. Therefore, we decided to present the PP analyses for the secondary outcomes.^39^ The software packages SPSS, version 27 (SPSS Inc) and StataSE, version 16 (StataCorp LLC) were used for statistical analysis. The significance threshold was *P* < .05.

## Results

### Study Population

Among the 521 participants examined by the study nurses at baseline, the mean (SD) age was 83.5 (6.17) years, 356 were women (68.3%), and 165 were men (31.7%). Ethnicity data were not collected because no scientific definitions are available for the population in Germany. A comparative analysis showed that there were no statistically significant differences between the populations in the intervention (n = 272) and control (n = 249) arms. Table 1 provides further baseline characteristics.

**Table 1.** COFRAIL Baseline Characteristics

Characteristic	No. (%)	
	Intervention	Control
**Study physicians**		
No. of practices (clusters)	56	54
No. of GPs	59	54
Sex		
Female	32 (54.2)	29 (53.7)
Male	27 (45.8)	25 (46.3)
Age, mean (SD), y	50.47 (8.39)	52.12 (7.94)
Years in general practice, mean (SD), y	13.30 (8.71)	13.75 (9.08)
Employee status		
Self-employed	48 (81.4)	45 (83,3)
Employed	11 (18.6)	9 (16.7)
Practice form		
Single	29 (51.8)	30 (55.6)
Group (≥2 GPs)	27 (48.2)	24 (44.4)
**Participants**		
No.	272	249
Sex, No.	272	249
Female	181 (66.5)	175 (70.3)
Male	91 (33.5)	74 (29.7)
Age, No.	272	249
Mean (SD), y	83.69 (6.08)	83.29 (6.29)
Educational level (CASMIN), No.^40^	269	242
Low	187 (69.5)	168 (69.4)
Medium	55 (20.4)	44 (18.2)
High	27 (10.0)	30 (12.4)
Clinical Frailty Scale, No.^35^	268	239
5 (Mild)	137 (51.1)	123 (51.5)
6 (Moderate)	102 (38.1)	90 (37.7)
7 (Severe)	29 (10.8)	26 (10.9)
No. of diagnoses^a^	266	249
Mean (SD)	11.77 (4.11)	12.11 (4.12)
Median (IQR)	11 (9-14)	12 (9-15)
Hospitalizations (6 mo before baseline), No.^b^	272	249
Mean (SD)	0.48 (0.85)	0.55 (0.82)
Median (IQR)	0 (0-1)	0 (0-1)
Medications, No.	270	246
Mean (SD)	9.28 (3.78)	9.37 (3.40)
Median (IQR)	9 (7-11)	9 (7-11)
Geriatric Depression Scale level, No.^41^	258	232
Normal	200 (77.5)	171 (73.7)
Mild to moderate	43 (16.7)	54 (23.3)
Severe	15 (5.8)	7 (3.0)
Barthel Index (functional restriction)^42^	272	247
U50.00 (none or low)	48 (17.6)	30 (12.1)
U50.10 (slight)	118 (43.4)	113 (45.7)
U50.20 (medium)	72 (26.5)	72 (29.1)
U50.30 (moderate)	21 (7.7)	17 (6.9)
U50.40 (severe)	10 (3.7)	9 (3.6)
U50.50 (very severe)	3 (1.1)	6 (2.4)
Care dependency category, level, No.^c^	270	247
No defined care level	48 (17.8)	42 (17.0)
1 (Low)	28 (10.4)	23 (9.3)
2	114 (42.2)	102 (41.3)
3	67 (24.8)	59 (23.9)
4	9 (3.3)	14 (5.7)
5 (Very severe)	3 (1.1)	3 (1.2)
No answer/not specified	1 (0.4)	4 (1.6)
Use of emergency services (at least once in the past 6 mo)^d^	271	246
Yes	31 (11.4)	24 (9.8)
Blood pressure, mm Hg, No.^a^	260	252
Systolic, mean (SD)	131.32 (17.85)	132.98 (16.28)
Diastolic, mean (SD)	75.52 (9.75)	75.64 (9.21)
Heart rate, min, No.^a^	236	239
Mean (SD)	72.92 (9.70)	71.86 (9.72)
Fasting glucose serum, mg/dL, No.^a^	244	227
Mean (SD)	85.41 (59.48)	78.74 (56.08)
GFR, No.^a^	253	238
Mean (SD)	55.04 (19.62)	53.87 (19.17)

Abbreviations: CASMIN, comparative analysis of social mobility in industrial nations; GFR, glomerular filtration rate; GPs, general practitioners.

SI conversion: To convert glucose to millimoles per liter, multiply by 0.0555.

^a^ Study physician's statement from the patient file.

^b^ Maximum of physician's indication and participant's indication.

^c^ Participants’ need for care assessed by the medical service of the German social care insurance.

^d^ Information obtained from participants/relatives.

The study population initially included 623 participants but decreased to 521 participants after 41 individuals in the intervention group and 61 in the control group dropped out before the study nurses were able to conduct the home visits for the baseline assessment. Of the 521 participants at baseline, a total of 129 individuals did not participate in T2 (65 in the intervention group, 64 in the control group). Reasons included withdrawal of consent (intervention, 16; control, 18), death (intervention, 29; control, 30), or other reasons (intervention, 20; control, 16).

For the ITT analysis, the primary outcome (number of hospitalizations) was also collected for the participants lost to follow-up at the time of dropping out. However, these data could not be obtained for 11 individuals, resulting in an ITT population of 510 participants.

A total of 385 participants (intervention, 200; control, 185) had a complete follow-up survey spanning 12 months (T0-T2) and attended at least 2 family conferences in the intervention group. These participants were included in the PP analysis (Figure).

**Figure.**
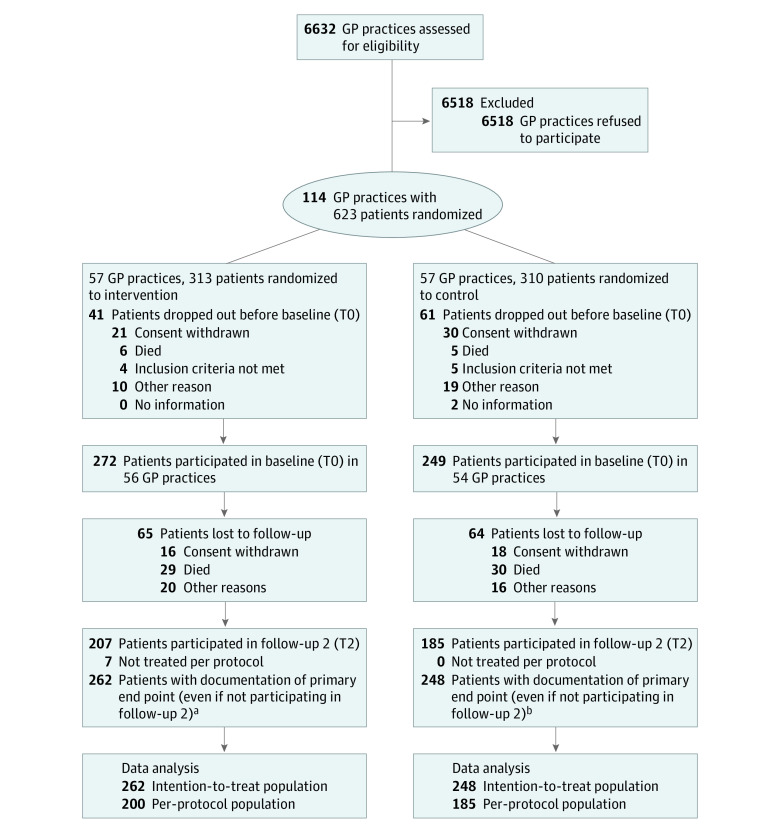
Consolidated Standards of Reporting Trials Diagram of Participant Flow Through Study GP indicates general practitioner. ^a^For 55 participants lost to follow-up, the primary outcome data were collected at dropout time. ^b^For 63 participants lost to follow-up, the primary outcome data were collected at dropout time.

In the ITT population, the mean (SD) observation time in the intervention group was 369.81 (87.61) days, compared with 335.58 (88.77) days in the control group. In the PP population, the mean observation time in the intervention group was 399.44 (45.58) days, compared with 373.72 (31.28) days in the control group.

To recruit the GPs, 6632 primary care practices were gradually contacted by post or email. Of these, 3.6% signed a consent form and 1.7% ultimately participated in the study. At baseline, 110 practices were included in the study (mean [SD], 4.74 [1.62] participants; median, 5 [IQR, 4-6] participants) at the 2 study centers, Düsseldorf (n = 68) and Rostock (n = 42).

All physicians in the intervention group attended the 2 mandatory training sessions. A total of 676 family conferences took place; 185 participants had 3 family conferences, 46 participants had 2, and 29 participants had 1 family conference during the intervention period.

### Primary Outcome

The ITT analysis of the hospitalization rate after 12 months included 510 participants. Due to the pandemic situation, the mean observation time was longer in the intervention group; therefore, an adjustment was made for the duration of observation. There was no significant difference in the adjusted mean (SD) number of hospitalizations between the intervention group (0.98 [1.72]) and the control group (0.99 [1.53]). Other results for the primary outcome are summarized in Table 2. There were no significant differences, even after adjusting the results regarding the observation period (mixed model 1) or other variables (mixed model 2), such as observation period, age, sex, number of chronic diseases, and retrospective hospital admissions in the 6 months before baseline. The analysis of the PP population of 385 participants yielded similar results.

**Table 2.** Primary Outcome: Mean Number of Hospitalizations Between Baseline and T2

Study participants	No. of hospitalizations				Mixed model 1		Adjusted mean No. of hospitalizations^a^				Mixed model 2^b^	
	Intervention group		Control group		IRR (95% CI)	*P* value	Intervention group		Control group		IRR (95% CI)	*P* value
	Mean (SD)	Median (IQR)	Mean (SD)	Median (IQR)			Mean (SD)	Median (IQR)	Mean (SD)	Median (IQR)		
ITT (n = 510)	0.86 (1.24)	0 (0-1)	0.79 (1.04)	0 (0-1)	1.08 (0.85-1.37)	.55	0.98 (1.72)	0 (0-1.19)	0.99 (1.53)	(0-1.58)	1.08 (0.84-1.39)	.53
PP (n = 385)	0.81 (1.21)	0 (0-1)	0.79 (1.10)	0 (0-1)	1.01 (0.79-1.31)	.91	0.73 (1.12)	0 (0-0.98)	0.79 (1.11)	0 (0-1.02)	1.09 (0.83-1.43)	.55

Abbreviations: IRR, incidence rate ratio; ITT, intention-to-treat analysis; PP, per-protocol analysis.

^a^ Adjusted for an observation period of 12 months.

^b^ Adjusting variables: length of observation period, age, sex, number of chronic diseases, retrospective number of hospitalizations at baseline.

Mixed-effect model Poisson regression, practices were taken into account as a random effect.

### Secondary Outcomes

The evaluation of the secondary outcomes was conducted for the PP population. The mean (SD) number of medications taken in the control group at T0 was 9.24 (3.44) per patient and 9.32 (3.59) at T1. In comparison, participants in the intervention group were taking a mean (SD) of 8.98 (3.56) medicines at T0. The number of medicines per patient decreased to 8.11 (3.21) at T1 and remained almost constant at 8.49 (3.63) at T2. At T1, a statistically significant difference between the groups was demonstrated (incidence rate ratio [IRR], 0.88; *P* < .001), but at T2 the difference did not reach statistical significance (IRR, 0.94; *P* = .07) (Table 3 and Table 4).

**Table 3.** Secondary Outcomes: Medications at Baseline and After 6 and 12 Months in the Per-Protocol Population

Data collection	Intervention group			Control group			Mixed model 1, IRR (95% CI)^a^	*P* value	Mixed model 2, IRR (95% CI)^b^	*P* value
	No.	Mean (SD)	Median (IQR)	No.	Mean (SD)	Median (IQR)				
Baseline	198	8.98 (3.56)	9 (6-11)	184	9.24 (3.44)	9 (6-11)	NA	NA	NA	NA
After 6 mo	193	8.11 (3.21)	8 (6-10)	181	9.32 (3.59)	9 (7-11)	0.88 (0.82-0.95)	<.001	0.88 (0.83-0.95)	.001
After 12 mo	197	8.49 (3.63)	8 (6-11)	184	9.16 (3.42)	9 (7-11)	0.94 (0.88-1.01)	.07	0.94 (0.88-1.01)	.08

Abbreviation: IRR, incidence rate ratio.

^a^ Mixed-effect Poisson regression model adjusted for the number of medications at baseline. Practices were taken into account as a random effect.

^b^ Practices were taken into account as a cluster or random effect. The adjusting variables included observation period, number of medications at baseline, age, gender, and number of chronic diseases.

**Table 4.** Secondary Outcomes: EU(7)-PIMs at Baseline and After 6 and 12 Months in the Per-Protocol Population^a^

Data collection	Intervention group			Control group			Mixed model 3, IRR (95% CI)^b^	*P* value
	No.	Mean (SD)	Median (IQR)	No.	Mean (SD)	Median (IQR)		
Baseline	176	1.57 (1.15)	1 (1-2)	171	1.80 (1.16)	2 (1-3)	NA	NA
After 6 mo	176	1.30 (1.05)	1 (1-2)	171	1.71 (1.25)	2 (1-2)	0.84 (0.70-0.99)	.04
After 12 mo	176	1.45 (1.21)	1 (1-2)	171	1.64 (1.15)	2 (1-2)	0.97 (0.82-1.15)	.72

Abbreviations: EU(7)-PIM, European Union list of the number of potentially inappropriate medication; IRR, incidence rate ratio.

^a^ The EU(7)-PIM for older people.^10^

^b^ Mixed-effect Poisson regression model adjusted for the number of EU(7)PIMs at baseline. Practices were taken into account as a random effect.

The application of a mixed regression model (model 1 mixed-effect Poisson regression) with adjustment for the number of medications at baseline (T0) as well as taking GP practices into account as a random effect resulted in a significant group difference between the intervention and control arms at T1 (IRR, 0.88; *P* < .001). However, this effect could no longer be detected at T2 (IRR, 0.94; *P* = .07). In mixed regression model 2, where age, sex, and number of diagnoses were additionally taken into consideration, there was also a significant group difference at T1 (IRR, 0.88; *P* = .001), which had decreased below the threshold of statistical significance at T2 (IRR, 0.94; *P* = .08).

After 6 months, the mean (SD) number of EU(7)-PIMs was significantly lower in the intervention group (1.30 [1.05]) than in the control group (1.71 [1.25]; *P* = .04). There was no significant difference in the mean number of EU(7)-PIMs after 12 months (Table 4). An additional exploratory analysis showed that the most commonly discontinued medications after 6 months were proton pump inhibitors, urate-lowering medications, statins, and oral antidiabetic agents.

The results for the assessments and safety parameters after 12 months are reported in the eTable in Supplement 2. For all results of these parameters, there were no statistically significant differences between the intervention and control group.

During the course of the study, a total of 59 (11.3%) of the 521 participants examined at baseline died within the 12-month observation period (intervention, 29 of 272 [10.7%]; control, 30 of 249 [12.0%]). There was no statistically significant difference between the groups with regard to mortality (*P* = .62; χ^2^ test).

## Discussion

In this pragmatic cluster randomized clinical trial, family conferences led by GPs in primary care were promoted to facilitate deprescribing and shared prioritization in community-dwelling older adults with frailty taking 5 or more medications. There were no significant differences between the intervention and control groups in the number of hospitalizations after 12 months as a measure of patient safety. This is in line with a number of other deprescribing studies in older adults with polypharmacy that have noted no positive impact on hospital admissions or other clinical outcomes.^16,43,44,45^ This finding is disappointing, as polypharmacy and EU(7)-PIMs are associated with adverse clinical outcomes,^10^ and a reduction in the number of daily drugs and EU(7)-PIMs should result in a decrease in adverse outcomes. On the one hand, the intervention effect assumed for our study might have been too large, so that considerably larger study populations would be required for future studies on the effect of deprescribing on hospitalizations. On the other hand, more appropriate cross-disease clinical end points would need to be identified that are both relevant and sensitive to change. In general, it is difficult to translate the impact of discontinuing drugs directly into clinical outcomes, as the effect of deprescribing, for example, unnecessary urate-lowering therapy or proton pump inhibitors over 6 or 12 months, may not have such a large influence on adverse drug-related effects resulting in hospitalization. However, given the risks associated with unnecessary polypharmacy, even small reductions in the overall drug burden may have a downstream effect on more subtle adverse effects not measured in our study or other comparable studies.

Regarding the secondary outcomes of our study, there was no significant difference between the intervention and control group in the number of medications taken per patient and in the number of EU(7)-PIM drugs according to the EU(7)-PIM list after 12 months in the PP population. These results are in line with many studies on deprescribing, which showed that even a reduction in medication in the first step (before clinical end points are realized in the second step) is not easy to achieve.^14,15,44,45^

However, the initial significance of changes in medication at 6 months, followed by nonsignificant changes at 12 months, suggests that it is difficult to sustain efforts to reduce medication use over time. Possible reasons for the convergence of prescription rates at 12 months for the most relevant drugs in our study (EU[7]-PIMs, proton pump inhibitors, urate-lowering medications, statins, oral antidiabetics) could be a recurrence of clinical problems that originally led to the prescription, represcribing of the medication by other medical specialists, and new clinical problems.

### Limitations

This study has limitations. The statistical power of the trial was limited because we were only able to include 510 participants in the ITT analysis of the primary outcome instead of the 608 we had originally planned. This was due to a smaller number of recruited participants and a higher dropout rate than expected. However, based on the available results, it can be assumed that even if the target number of individuals had been reached, there would not have been a chance to show a statistically significant difference in the primary end point.

In addition, the limited statistical power results in a lower certainty to detect a statistically significant difference between the intervention and control group in the other direction and to exclude a harmful effect of the COFRAIL intervention. Another limitation is that comprehensive information about the reasons for each hospital admission could not be collected in our study setting.

The analyses of the secondary end points were performed for the PP population and not for the ITT population due to the lack of measurement options for the dropout participants, which could lead to an overestimation of the observed effects. The switch in data collection by the study nurses from home visits to telephone interviews because of the COVID-19 pandemic may have contributed to comparatively high dropout rates and led to limitations in data quality.

At the patient level, it is possible that the GPs included more patients in whom they expected to see a high level of adherence to the study, which limits the representativeness of the study. To minimize selection bias, neither the study physicians nor the patients were informed about the intervention elements at the time of recruitment.

## Conclusions

In this cluster randomized clinical trial with older adults taking 5 or more medications, the intervention consisting of GP-led family conferences did not achieve sustainable effects in reducing the number of hospitalizations or the number of medications and EU(7)-PIMs after 12 months. From the transient reduction in the number of medications and EU(7)-PIMs achieved by the COFRAIL intervention at 6 months, it can be concluded that family conferences for shared decision-making can successfully initiate the process of deprescribing.

## Notes

Supplement 1.
Trial Protocol and Statistical Analysis Plan

Supplement 2. **eTable.** Geriatric Assessments and Safety Parameters at Baseline (T0) and After 12 Months (T2)
eReferences

Supplement 3.
Data Sharing Statement
