# Supplementary material for: Family Conferences to Facilitate Deprescribing in Older Outpatients With Frailty and With Polypharmacy: The COFRAIL Cluster Randomized Trial
Source: JAMA Netw Open. 2023 Mar 27;6(3):e234723. doi: 10.1001/jamanetworkopen.2023.4723 (PMC10043750; doi:10.1001/jamanetworkopen.2023.4723)
Supplement: Supplement 1. — Trial Protocol and Statistical Analysis Plan [file jamanetwopen-e234723-s001.pdf]

Ethics application - non AMG* study

1. Basic data

1.1 Full title of the study.

Family conferencing in frailty: increasing patient safety through shared prioritization (Cofrail).

1.2 Principal investigator at the local study center.

Attila Altiner, Facharzt für Allgemeinmedizin, Prof. Dr. med., Institut für Allgemeinmedizin, Universitätsmedizin Rostock, Doberaner Str. 142, 18057 Rostock, Tel.: 0381/ 494 2481, Fax: 0381/ 494 2482, Email: attila.altiner@med.uni-rostock.de

Anja Wollny, Gesundheitswissenschaftlerin, Dr. phil, Institut für Allgemeinmedizin, Universitätsmedizin Rostock, Doberaner Str. 142, 18057 Rostock, Tel.: 0381/ 494 2484, Fax: 0381/ 494 2482, Email: anja.wollny@med.uni-rostock.de

1.3 Background of the study

The study focuses on geriatric frailty syndrome. This refers to a state of physical frailty with increased predisposition to the development of dependency and morbidity in older persons. Frailty is often the result of the presence of multiple diseases or multiple functional limitations and includes the following characteristic features: Weight loss, loss of muscle mass, fatigue, exhaustion, and reduced physical activity. Frailty is associated with an increased risk for the occurrence of falls and confusional states, as well as hospitalization and mortality. Geriatric frailty syndrome can be improved with interventions such as increased physical activity.

Polypharmacy is an independent risk factor for the occurrence of falls, hospitalization, and increased mortality, and is considered a significant pathogenetic factor for the occurrence of geriatric frailty syndrome. However, polypharmacy is also a risk factor for overuse of inadequate medications. Reducing polypharmacy (deprescribing) thus represents a promising intervention for improving or stabilizing the health status of frailty patients.

Care for patients with frailty syndrome in the outpatient sector is often difficult due to the complexity of the problems. Lack of care coordination in turn promotes unplanned hospital admissions as a result of fall events, pain syndromes, confusion or weakness. Since hospitalization is costly for these vulnerable patients and is itself a high risk, reducing hospitalization rates is an important goal of primary care case management in geriatric frailty syndrome. This should be achieved by strengthening the empowerment of patients and their relatives. The challenge of patients with frailty syndrome for family physicians comprises not only a better organization of primary care, but above all the prioritization of possible therapeutic measures based on the individual preferences of the patient together with the patient, the family caregivers and, if necessary, the professional carer. Following on from this, family conferences

** Medicinal Products Act (Arzneimittelgesetz – AMG)*

will be used in the study as a potentially effective tool for the care of patients with frailty syndrome in family practice.

1.4 Objective and aim of the study

The study investigates the effectiveness of family conferences (patient, general practitioner, family member, nursing service) in the context of a complex intervention with regard to patient-related outcomes in elderly patients with geriatric frailty syndrome and polypharmacy. For this purpose, we will examine whether the intervention leads to a reduction in the average number of hospitalizations per patient (hospitalization rate) compared to the control.

Second, we will investigate whether the intervention compared to the control:

1) to an improvement of the patients' health status regarding the expression of the frailty syndrome, cognition, quality of life, activities of daily living,

2) to a reduction in the frequency of prescription of potentially inadequate medications (PIM),

3) leads to better consideration of individual patient preferences and existing family resources from the perspective of patients, family members, and primary care physicians, and

4) proves to be cost-effective in terms of achieving the above goals.

The study is conducted as a pragmatic cluster randomized controlled trial (cRCT).

1.5 on-site institutes/clinics/practices involved in the study (address, telephone)

none

1.6 How is the study financed? Cost unit? sponsor?

Innovationsausschuss beim Gemeinsamen Bundesausschuss
Funding code at the Innovation Committee: 01VSF17053
Consortium management: Institute of General Medicine, University Hospital Düsseldorf

1.7 Is this a multicenter study?

Yes

Recruitment and data collection will be conducted at two sites (Düsseldorf and Rostock).

The Institute of Health and Nursing Science of the Medical Faculty of the Martin Luther University Halle-Wittenberg is responsible for the process evaluation (for details on the process evaluation, see 2.4).

1.8 Voting of other ethics committees

Has an application with the same content already been submitted to another ethics committee in Germany?

No

1.9 Which special laws/regulations do you have to observe for your study?

Federal Data Protection Act (version of the BDSG of 25.05.2018 according to EU directives) and Basic Data Protection Regulation (DSGVO)

1.10 Information on economic and other conflicting interests of the study management in connection with the study.

There are no conflicts of interest.

2. Summary characterization of the study

2.1 Type of test

Other, namely: Testing the effectiveness of a complex intervention.

2.2 Study design

Pragmatic cluster-randomized controlled trial (cRCT) with complex intervention (see Figure 1: Study design).

2.2.1 Methods

This is a cluster-randomized controlled intervention study. Cluster randomization is performed at the level of primary care practices.

Recruitment and randomization
A total of 136 GPs in Rostock (n=46) and Düsseldorf (n=90) will be recruited. Subsequent recruitment of the total of 676 patients will be performed by the GPs in the practices based on the defined inclusion and exclusion criteria (Rostock n= 228, Düsseldorf n= 448). After the baseline survey, the GPs will be randomized into two arms (intervention and control). The complex design of the intervention does not allow blinding.

Control
In the primary care physicians belonging to the control group, none of the intervention measures listed below take place; instead, the standard care (care as usual) is observed and further medical education on the topic of geriatrics is offered.

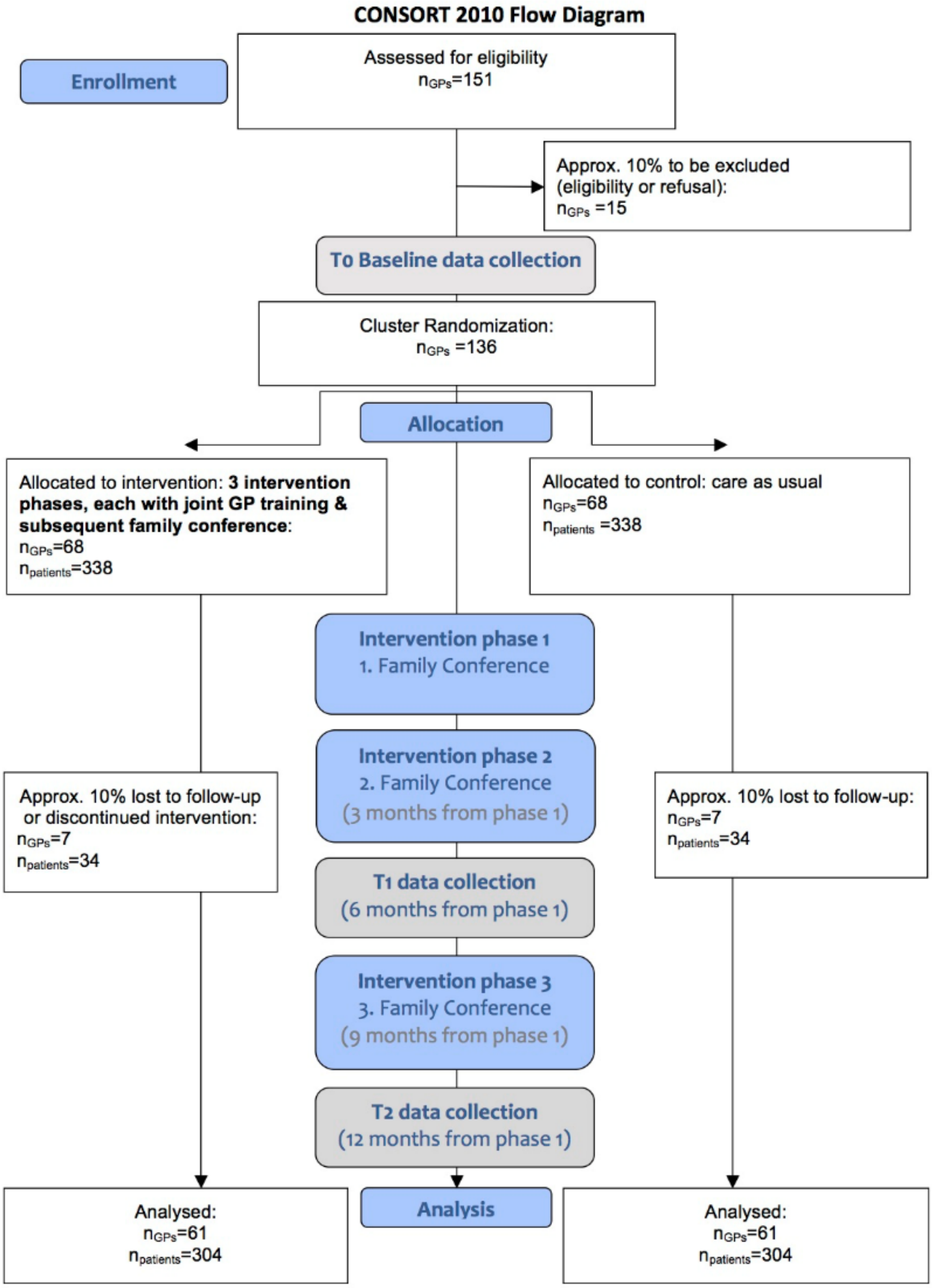

Figure 1 - Flow chart of the study

Intervention

The intervention will be conducted in two phases:

Phase 1 (preparation of the intervention): The participating primary care physicians of the intervention group will receive three consecutive training sessions, in which medical knowledge as well as competencies for the implementation of structured family conferences will be trained by means of communication training (2 obligatory face-to-face sessions, 1 optional training session, possibly as a webinar). In this context, physicians are provided with evidence-based specialist information as well as written materials to support the discussion with patients and relatives, including a toolkit with relevant non-pharmacological interventions. Obligatory content is a medication check with joint prioritization of drug therapy in the context of the family conference. Family physicians can optionally request an individual medication review by a clinical pharmacologist via a hotline. Other optional elements of the toolkit include:

a) Consultation on measures to maintain mobility, fall prevention and pain therapy;

b) Joint preparation of an emergency plan, if necessary taking into account a living will;

c) Discussion of any existing need for nursing and psychosocial support, arrangement of nursing care counseling by a local nursing care support center.

Phase 2 (implementation of the intervention in the family practices): Conducting three family conferences per patient at baseline and at 3 and 9 months for 30-45 minutes each during a home visit involving family caregivers and/or care providers. The joint discussion should include:

a) first, the patient's general preferences regarding his or her health care are worked out (e.g., regarding possible indications for hospitalization),

b) a medication check is drawn up, in which the preferences initially worked out are applied,

c) further topics from the above-mentioned toolkit are worked on as required.

This is followed by a summary with documentation by the GP and an agreement on the further procedure, such as follow-up appointments.
The study period per patient is 12 months with data collection at T0 (baseline), T1 (6 months) and T2 (12 months).

Data collection
In all patients (of both study arms), several parameters will be collected or survey instruments will be used before the beginning (baseline), during (6 months) and at the end of the intervention (12 months):
Basic data (once before start and if necessary during the course)
1. age
2. gender
3. educational level
4. marital status
5. living situation

6. degree of care
7. degree of disability

At baseline (T0), during the intervention (T1), and after completing the intervention (T2).

- Average number and duration of hospitalizations per patient (to determine hospitalization rate).
- Relevant diagnoses
- weight history
- physical weakness (hand grip strength with dynamometer)
- subjective assessment of the degree of fatigue
- walking speed
- degree of physical activity
- cognition (episodic memory, executive function CERAD test)
- Number of medications/active ingredients taken, among others, to determine potentially inadequate medications
- Psychological status (Geriatric Depression Scale (GDS))
- Health-related quality of life (EQ-5D, VAS scale)
- frequency of falls
- Activities in daily living (Barthel index)
- blood pressure
- pulse
- Cardiovascular morbidity (myocardial infarction, apoplex)
- mortality
- Costs (incremental cost-effectiveness ratio)

2.3 Study participants

The recruitment procedure is multi-step. One after the other, first GPs and then patients will be recruited. A total of 676 patients will be included by 136 GPs (5 patients per GP).
During recruitment by the GP, the patient will be asked to name a relative as the main reference person, who will be present at the family conferences. If applicable, a representative of the ambulatory care service will also be invited to participate.

2.3.2 Inclusion and exclusion criteria:

GPs: can participate if they are registered as specialists in general medicine or specialists in internal medicine practicing general practice with the responsible Association of Statutory Health Insurance Physicians.
Patients: Inclusion is pssible if all of the following criteria are met:

1) Positive screening for geriatric frailty syndrome.
2) Age 70 years
3) Regular intake of 5 agents (polypharmacy).
4) Need for nursing care (nursing degree 1 or higher)
5) Need for Nursing care in their own home by family members and/or outpatient nursing service

Exclusion occurs when, in the judgment of the primary care physician, one or more of the following criteria are present:

1) moderate or severe dementia
2) reduced life expectancy of 6 months (palliative care)
3) nursing home residents
4) insufficient language skills in German of patient and family caregivers or non-availability of an interpreter

Relatives or trusted persons and professional carers: can participate if specified by the patient.Tthe patient will also be asked whether a professional carer should be involved in the family conferences.

Please justify the extent to which the selected gender distribution is appropriate for identifying possible gender differences (e.g., in the efficacy of the treatment, the safety of the medical device).
Female patients as well as male patients are consecutively included in the study without stratification to sex.
In the evaluation, gender is considered throughout as one of the relevant factors that may influence the results. In particular, for questions related to the outcome of care, potential differences for men and women will be described explicitly.

2.4 Process evaluation:

In order to be able to understand the change process and for the generalizability of the study results, a comprehensive collection of process data accompanying the pragmatic intervention study is essential. Process evaluation is conducted according to international recommendations for process evaluations of complex interventions on the topics: Implementation, Mechanisms of Action, and Context. It focuses on the following questions: what is implemented and how is it implemented (fidelity, dose, adaptation, reach)? How does the administered intervention lead to change (particpant response, mediators, unanticipated pathways and consequences)? How does context influence implementation, mechanisms, and outcomes?

Therefore, different process parameters on cluster (practice) and individual level (participants) will be collected with qualitative and quantitative methods (cf. Tab. 1). To investigate the feasibility and to optimize the intervention, semi-standardized interviews with family physicians and short telephone interviews with patients will be conducted after piloting the family conferences. The recruitment process, including reasons for nonparticipation, will be documented at both the cluster and individual levels. Contextual factors will be collected taking into account important structural and process-related aspects (e.g. socioeconomic and sociodemographic characteristics of GPs and patients) at baseline. To determine intervention fidelity, a structured documentation form will be completed by both school attendees and primary care physicians after each training session. In addition, the family conferences will be evaluated using a semi-standardized questionnaire at t0, after three months, and after six months, to be completed by primary care physicians immediately following the family conference.

The attitudes towards the COFRAIL intervention and the GPs' experiences with it will be discussed in four focus groups with a convenient sample of six to twelve participants at the end

of the study. In addition, semi-standardized individual interviews will be conducted with a random sample of approximately 10 GPs per region.
In a subsample of 10%, families' experiences (e.g., consideration of preferences; changes in physician-patient communication; barriers and facilitators) will be assessed after 12 months using semi-standardized individual interviews.
The process evaluation is exploratory only. All quantitative data are analyzed descriptively; all qualitative data are analyzed using qualitative content analysis according to Mayring. (All process evaluation documents regarding informed consent can be found in Annex 5-7).

Table 1: Content of the process evaluation.

**Focus**	**Data collection method/documentation**	**Date of data collection**
Feasibility of the intervention	Piloting of family conferences with two GPs/study center: semi-standardized interview with GPs; brief telephone interview with patients and relatives.	Piloting, before baseline
Recruitment process of GPs and patients	**Minutes**/Study center	t_0
Reasons for non-participation or drop out	**Structured query** and documentation of reasons	t_0-t_2
Description of key structure- and process-related factors at the cluster and patient levels.	**CRF-Baseline Data**/Cluster and patients	t_0
Intervention fidelity	Obligatory Continuing Education Units: **Structured protocol** for each training unit<br><br>Utilization of the optional training unit: **Standardized documentation**	t_0 (immediately after the training courses)
Effects of the educational intervention	All participants of the GP training program: **Standardized questionnaire** → Evaluation of the program<br>• Attitudes<br>• Acceptance<br>• Self-efficacy<br>• Expectations	t_0 (before and after the training courses)
Application of training content	All GPs: **Semi-structured protocols** → Evaluation of family conferences.<br>• Acceptance<br>• Contents<br>• Duration<br>• Feasibility<br>• Need for adaptation	t_0 , after 3 and 6 months (immediately after the family conferences).
GPs' experiences (e.g., attitudes toward the intervention; changes in doctor-patient communication; barriers and facilitators)	**Four focus groups**; two/study center with 6 to 12 GPs.<br><br>**Semi-standardized interviews**; random sample of 10 GPs/study center.	t_2
Patient experiences (e.g., consideration of preferences; changes in physician-patient communication; barriers and facilitators).	**Semi-standardized interviews;** 10% of participants/study center.	t_2

3. Protection and safety of the study participants.

3.1 and 3.2 Description of the procedure for recruiting of study participants and obtaining informed consent for study participation from study participants, parents or legal guardians, if applicable.

Recruitment method:

The recruitment procedure is multi-step. At two locations (Düsseldorf, Rostock) as many GPs will be contacted step by step until the necessary number is reached. In addition, GPs will be invited by mail to participate in a training course or in the study. In the next step, all interested GPs will be informed about the study verbally or in writing. After expressing interest, the signed consent form is obtained from the GPs. Subsequently, the general practitioner draws up a list of patients who are basically eligible for participation in the study on the basis of the billing code for geriatric diseases in the last three months (EBM code 03362). The general practitioner and the study assistant employed in the project jointly check which patients are potentially suitable for study participation on the basis of the inclusion and exclusion criteria (see 2.3.2). Subsequently, patients are contacted consecutively by the GP and their willingness to participate is requested until the number of five patients per GP is reached or the list is completed. The written consent of the patients is obtained by the GPs (consent form), after which the contact details are passed on to the local study center. After baseline data collection (t0), randomization into control and intervention group will be performed. The patients in the intervention group will be asked to name a caregiver/relative and, if necessary, a representative of the nursing service. The other participants will also be informed about the study.

All recruitment documents for patients and GPs can be found in the attachment to this application. These include cover and info letters for GP and patient, patient inclusion checklist or study flow chart (GP) and informed consent form (patient and GP). Furthermore, the information letters for caregivers/relatives and representatives of the nursing service are attached (see Annex 1-4).

Informed consent:

The GPs will be informed about the study in both written and verbal form by the local study center and will be requested to give written consent. Patients will be informed and educated in detail about the study and data protection regulations by the GPs, then if necessary by the study staff. Study participation and data collection are only permitted after the patients have given their consent. All patient information materials (cover letters, patient information letters and consent forms) and physician information are attached to this application (see Attachments 1 and 2).

Duration of recruitment (enclose materials such as advertisements, leaflets, etc.).

Recruitment of GPs will be done after submission of ethics vote from 01/12/2018 and will be completed within 6 months. From 01/04/2019, recruitment of patients will be done by GPs within four months.

3.3 Particularly vulnerable persons:

Are persons under the age of 18 participating in the study?	No
Do non-consenting adults participate?	No

3.4 Justification for the inclusion of vulnerable subjects
(such as healthy or ill minors, non-consenting adults) Why can't the study be performed on consenting adults?
Not applicable

3.5 Who will provide medical care to the study participants before, during and after the study?
(e.g. coordination with the GP? Control of other medications? Control of blood levels?)
Participating patients will be cared for by their GP before, during and after the study as usual. In case of withdrawal of consent by the patient, their treatment by their GP and, if necessary, nursing care will be assured in the same way as before.

3.6 Do the participating persons receive an reimbursement of expenses or a payment?
All participating physicians receive 200€ reimbursement per patient, regardless of their group assignment (intervention / control). The patients and their confidant or representative from the ambulatory care service do not receive any payment for their participation.

3.7 Has insurance (possibly also route insurance) been taken out for the study participants?
No

4 Documentation, evaluation, reporting

4.1 What type of documentation is planned? (Attach documentation forms if necessary).
The outcome variable of the primary research hypothesis is the reduction in the average number of hospitalizations per patient (hospitalization rate) within 12 months of the start of the intervention.
The secondary research hypothesis addresses the improvement of the patient's health status in terms of frailty syndrome expression (weight progression, physical weakness, fatigue level, walking speed, physical activity), cognition, number of medications taken, psychological status, health-related quality of life, fall frequency, activities of daily living, and costs.
In addition, relevant diagnoses as well as the safety parameters blood pressure, pulse, cardiovascular morbidity and prognosis related to mortality will be recorded.
The following table describes where and how the target parameters will be collected.

Table 2: Overview of data collections and sources

Research hypothesis	Item	Data source
Primary	Number/duration of hospital stays	GP data collection form; Patient data collection form
Secondary	Relevant diagnoses	GP data collection form
Secondary	Number of drugs taken, PZN	GP data collection form; Patient data collection form
Secondary / safety parameter	Blood pressure	GP data collection form
Secondary / safety parameter	Heart rate	GP data collection form
Secondary / safety parameter	Cardiovascular morbidity (myocardial infarction, apoplexy)	GP data collection form
Secondary / safety parameter	Prognosis/ mortality	GP data collection form
Secondary	Weight trend	Patient data collection form
Secondary	Physical weakness	Patient data collection form
Secondary	Degree of exhaustion	Patient data collection form
Secondary	Walking speed	Patient data collection form
Secondary	Degree of physical activity	Patient data collection form
Secondary	Cognition	Patient data collection form
Secondary	Psychological status	Patient data collection form
Secondary	Health-related quality of life	Patient data collection form
Secondary	Frequency of falls	Patient data collection form
Secondary	Activities of daily living	Patient data collection form

Secondary	Costs	Patient data collection form

Data flow:
Baseline data collection and data entry into a database (ORACLE database, see also section 4.4) will be performed by the study nurses of the local study centers (t0).
Six months and 12 months after baseline data collection, follow-up data collection (t1, t2) will be performed by the GPs and study nurses, and data entry into the database will be performed by the study nurses.

4.2 Is an external monitoring provided?
No

4.3 Is cooperation with a statistician planned?
Yes: Dipl.-Math. Birgitt Wiese, Institute of General Medicine, Hannover Medical School (MHH)

4.4 Measures provided for the protection of the collected data
The data will be entered into a central ORACLE database (Oracle 9i) at the local study centers (Düsseldorf and Rostock) using a web-based data entry system secuTrial®. Access to the internal database and the web server will be controlled by two firewalls connected in series. The data transfer will be done by 128 bit SSL encryption. There will not be a local data storage. secuTrial® fulfils the requirements of the FDA (21 CFR Part 11) and complies with the guidelines for Good Clinical Practice (GCP).
The data will be stored with a pseudonym without identification data. The members of the study group will have access to the electronic data entry system according to a detailed user role and rights concept. An audit trail ensures an automatic protocol of all data entries, changes and deletions. Various data quality assurance measures will be implemented: These include online plausibility checks during data entry, data checks after data entry and user training before data entry. In addition, all those involved in data collection and entry will be trained by the data management team (study assistants, student assistants) and will receive a manual describing and explaining all functions.

4.5 Have discontinuation criteria been defined?

- for the individual **study participants**: Yes
  - Participation can be withdrawn by individual patients at any time without giving reasons.
- for the termination of the **entire study**: Yes
  - The criterion for termination of the study is defined as an increase in the hospitalisation rate of > 30% in the control and/or intervention group.

5 Consideration of the harm/benefit potential

5.1 Harm and risks

What are the types of potential risks, harms, burdens or other disadvantages for the persons participating in the study?

The intervention aims to improve communication between GPs, patients and relatives. Medical risks are thus not to be expected, as the study does not directly intervene in medical and pharmacological treatment.

It is assumed that there is no risk or harm to be expected for the patients participating in the study. Nevertheless, in addition to the hospitalization rate, blood pressure, pulse and mortality are defined as safety parameters, which will be regularly analyzed by the project administration and by the cooperation partners.

For data collection purposes, patients in both the intervention and control groups will be interviewed by trained study assistants. This will only be done with the explicit consent of the patients. In addition, the study assistants will be trained by the study team so that they are prepared to interact with older people. The patients will be given sufficient time to answer the questions or carry out the tests, as these could be perceived as strenuous. Breaks/interruptions of the interviews will be offered if needed.

5.2 To what extent, if any, do study-related measures deviate from the usual routine treatment?

All patients will receive the usual routine treatment within the framework of normal care by their participating doctors. Nursing services will continue to be provided by the outpatient nursing service as before.

In the intervention group we additionally offer:

Conducting three family conferences per patient at the beginning of the study as well as after 3 and 9 months for 30-45 minutes each in the context of a home visit involving the family caregiver and/or a professional caregiver. The joint conversation should include the following:

- First, the patient's general preferences regarding his or her health care will be identified (e.g. possible indications for hospitalisation),
- a medication check will be conducted, in which the initially elaborated preferences will be applied,
- If appropriate, further topics from the toolbox (non-pharmacological interventions to secure mobility, emergency planning, provision of psychosocial support) are dealt with and a summary of the further procedure is prepared by the GP.

(see paragraph 2.2.1 Intervention).

5.3 Benefits and opportunities

What is the expected benefit?

Individual Benefit: Improving drug therapy safety (AMTS) with a reduction in the number of potentially inappropriate medicines (PIM) prescribed and with a reduction in drug-related side effects (fall rate, hospitalisations) contributes to patient safety. In addition, an improvement of the health status compared to the control group is intended through a reduction of the expression of the frailty syndrome in the course. Furthermore, the participation and self-determination of patients with frailty syndrome will be improved by strengthening shared prioritisation and participatory decision-making by participating in the family conference.

Benefits for care: The aim of the family conference is to improve the communicative skills of GPs in dealing with complex treatment problems. This method will improve communication and cooperation between the actors involved. This enables a more structured understanding of problems and the targeted search for solutions. Patients who do not directly participate in the study may also benefit from this.
External benefits: The knowledge gained could be used to discuss the use of the family conference instrument for other complex areas of primary care. If the evaluation is positive, these results can be incorporated into the development of didactic formats for continius medical education and undergraduate training of physicians.

5.4 Please justify why you consider the potential harm to be justifiable in relation to the expected benefits.

In the context of improving patient safety, harm can almost be excluded. Patient participation is voluntary and consent can be withdrawn at any time without any disadvantages. By connecting the competences of the professional groups caring for the patient, a more efficient solution can be ensured in the in case of a problem arising.

6. Signatures and statement

6.1 Statement and signature of the director of the clinic or institute (with stamp)

I agree to the conduct of the study mentioned under 1.1. I confirm that the resources (number and qualification of staff, infrastructure, equipment and facilities) for a successful implementation are available.
I also confirm that the recruitment of study participants will not be compromised by competing studies.

(Stamp) Rostock, Date
Signature (Prof. Dr. Attila Altiner)

Annex

1 - Recruitment documents GPs (cover letter, study information, fax response, consent form).
2 - Recruitment documents patients (cover letter, study information, consent forms)
3 - Study information to caregiver/relatives,
4 - Study information for nursing staff
5 - Process evaluation documents for general practitioners
6 - Documents process evaluation patients
7 - Process evaluation documents for caregivers/ relatives

COFRAIL - Statistical analysis plan

1. Introduction

The COFRAIL study is a multicenter cluster-randomized controlled intervention trial to increase patient safety through promoting family conferences in primary care.

2. Data source

676 patients will be recruited in 138 general practices, with the practices randomised into the intervention and control groups in a ratio of 1:1, so that 338 patients will be assigned to the intervention group and 338 patients to the control group. Data will be collected at baseline T0, at T1 (after 6 months) and at T2 (after 12 months). In addition, in the intervention group, data are collected on three family conferences that take place between T0 and T1 or between T1 and T2. Sociodemographic data, data on quality of life (EQ-5D-5L), memory tests (CERAD), physical functionality, geriatric depression scale, medication, hospital stays, doctor contacts, history of falls, as well as other data relevant to health economics will be collected. The data will be entered into a central ORACLE database in the web-based EDC system secuTrial®. In order to achieve high data quality, extensive plausibility checks are implemented in the input system; in addition, further data checks will be conducted after the data has been entered.

3. Objective, endpoints and statistical analyses

The main objective criterion - the primary outcome - is the number of hospitalisations per patient within the observation period of 12 months.

The secondary outcomes are the following:

- Medication: number of medications per patient, Drug Burden Index (DBI) defined as number of anticholinergic or sedative medications and prevalence of potentially inadequate medication.
- Hand force measurement
- Cognition: scores of the CERAD tests "word list" and "naming animals
- Sum score of the Geriatric Depression Scale (GDS)
- Mobility measured by the Timed Up & Go - Test
- Health-related quality of life (EQ-5D-5L)
- Activities of daily living measured by the Barthel Index
- Weight

The statistical analyses will include detailed descriptive statistics (frequencies, position and variance parameters) for the parameters (total and separately for the intervention and the control group) to describe the study population (age, gender, education according to CASMIN, frailty index, etc.) as well as to assess the primary outcome and the secondary endpoints.
A mixed regression model will be used for the analysis of the primary outcome. As a random effect, the practice affiliation characterized by the practice ID will be included in the model. In addition to the group (control or intervention), the factors age, gender and co-morbidities are

included as fixed effects for adjustment. A linear mixed model with the target variable "number of hospitalizations" is envisaged; according to the actual distribution of the target variables, an ordinal logistic or a Poisson model can also be applied.

The secondary outcomes will be analyzed using mixed linear regression models or mixed logistic regression models (binary or ordinal) depending on the distribution. The practice affiliation is included in the model as a random effect, fixed effects are group affiliation, age, gender and comorbidities.

As an alternative to the classical regression methods, the methods of Classification Tree Analysis (CART) will be applied. This method focuses on subgroups of cases that are as homogeneous as possible with regard to the distribution of the response. The characterisation of homogeneous subgroups is done with the help of a binary tree: first, the entire sample will be split into two subspaces. Each subspace created in this way and all further subspaces can be successively split further so that a hierarchically structured division will be obtained overall. The CART analysis is carried out with 10-fold cross-validation. The cut-points of the predictors are determined by the procedure; missing values in the predictors are replaced by surrogate variables. Target parameters in the respective CART analyses are the outcomes (primary and secondary); predictors are group membership (control or intervention), age, gender, co-morbidities and other possible influencing factors. Advantages of these methods are the independence of the distribution of the predictors as well as the easily interpretable presentation of the results as a decision rule.

The evaluation of the safety parameters (in particular mortality and hospitalizations) will be provided to the Data Safety Monitoring Board (DSMB) at regular intervals descriptively as frequencies blinded (i.e. it is not recognizable which group represents the intervention and which the control).

4 Aim, outcomes and statistical analyses of the health economic evaluation

The aim of the health economic evaluation is to determine the efficiency of the intervention by comparing the costs and outcomes of the intervention group with the costs and outcomes of the control group (care as usual). All costs related to the intervention as well as to the utilisation of health care services (Krauth et al., 2005) from the perspective of social insurance in Germany (health insurance, long-term care insurance and pension insurance) will be considered.

To determine the efficiency of the intervention, a cost-effectiveness analysis and a cost-utility analysis will be performed. The incremental cost-effectiveness ratio (ICER: additional costs for each additional hospital admission avoided) and the incremental cost-utility ratio (ICUR: additional costs for each additional quality-adjusted life year (QALY) gained) will be calculated as a quotient of the cost and benefit differences between the intervention group and the control group. QALYs are utility values and are calculated by recording health-related quality of life with an established preference-based quality of life instrument based on the EQ-5D-5L (Herdman et al., 2011) and economically valued with a German health cost table (Greiner et al., 2005) to generate utility values.

95% confidence intervals for outcome and costs will be determined non-parametrically based on distributional characteristics using bootstrap procedures (Briggs, 1997). Univariate and probabilistic sensitivity analyses will be performed to account for uncertainty, and cost-effectiveness acceptability curves will be constructed (Fenwick, 2004).

5. Analysis population

The evaluation of the primary outcome measure will follow the intention to treat (ITT) principle. This means that all patients who are included in the study, i.e. all intended patients in the intervention group and all patients in the control group who give their study consent, will be included in the analysis. The patients remain in the original group for the analysis, even if, for example, the family conferences are not carried out for a patient in the intervention group. If the study is terminated before completion, the number of hospitalisations up to the time of termination is used as the primary endpoint, i.e. the Last Observation Carried Forward (LOCF) principle is applied.

As a sensitivity analysis, a per protocol (PP) analysis will be performed, i.e. all patients who properly completed the study will be included in this analysis.

6. Data processing

Missing values will not be replaced a priori. Some of the procedures mentioned above (CART, RPA) can handle missing values, i.e. missing values are replaced by so-called surrogate variables only for the split criteria. Sensitivity analyses will be carried out to investigate possible biases due to missing values that are not missing by chance.

7. Software used

The analyses will be carried out with the software packages SPSS, STATA, SAS, and CART.

8. References

Breiman L, Friedman JH, Olshen RA, Stone CJ. Classification and Regression Trees. Chapman & Hall (Wadsworth, Inc.): New York, 1984.

Briggs, A.H., D.E. Wonderling, and C.Z. Mooney, Pulling cost-effectiveness analysis up by its bootstraps: a non-parametric approach to confidence interval estimation. Health economics, 1997. 6(4): p. 327–340.

Fenwick, E., B.J. O'Brien, and A. Briggs, Cost-effectiveness acceptability curves--facts, fallacies and frequently asked questions. Health economics, 2004. 13(5): p. 405–415.

Greiner W, Claes C, Busschbach JJ, von der Schulenburg JM. Validating the EQ-5D with time trade off for the German population. The European journal of health economics : HEPAC : health economics in prevention and care. Jun 2005;6(2):124-130.

Herdman, M., et al., Development and preliminary testing of the new five-level version of EQ-5D (EQ-5D-5L). Qual Life Res, 2011. 20(10): p. 1727-36.

Krauth, C. et al. (2005). Empirical standard costs for health economic evaluation in Germany- a proposal by the working group methods in health economic evaluation. Gesundheitswesen, 67(10), 736-746.

Ethikantrag – nicht AMG Studie

1. Basisdaten

1.1 Vollständiger Titel der Studie

Familienkonferenzen bei Frailty: Erhöhung der Patientensicherheit durch gemeinsame Priorisierung (Cofrail)

1.2 Studienleiter/Studienleiterin vor Ort

Attila Altiner, Facharzt für Allgemeinmedizin, Prof. Dr. med., Institut für Allgemeinmedizin, Universitätsmedizin Rostock, Doberaner Str. 142, 18057 Rostock, Tel.: 0381/ 494 2481, Fax: 0381/ 494 2482, Email: attila.altiner@med.uni-rostock.de

Anja Wollny, Gesundheitswissenschaftlerin, Dr. phil, Institut für Allgemeinmedizin, Universitätsmedizin Rostock, Doberaner Str. 142, 18057 Rostock, Tel.: 0381/ 494 2484, Fax: 0381/ 494 2482, Email: anja.wollny@med.uni-rostock.de

1.3 Hintergrund der Studie

Die Studie fokussiert auf das geriatrische Frailty-Syndrom. Dieses bezeichnet einen Zustand körperlicher Gebrechlichkeit mit erhöhter Anfälligkeit für die Entwicklung von Pflegebedürftigkeit und Morbidität bei älteren Personen. Frailty ist häufig das Resultat des Vorliegens multipler Krankheiten oder mehrerer Funktionseinschränkungen und umfasst folgende charakteristische Merkmale: Gewichtsabnahme, Verlust an Muskelmasse, Müdigkeit, Erschöpfung und reduzierte körperliche Aktivität. Frailty ist mit einem erhöhten Risiko für das Auftreten von Stürzen und Verwirrtheitszuständen sowie für Hospitalisierung und Mortalität verbunden. Das geriatrische Frailty-Syndrom kann durch Interventionen wie z.B. erhöhte körperliche Aktivität verbessert werden.

Polypharmazie ist ein unabhängiger Risikofaktor für das Auftreten von Stürzen, Krankenhausbehandlungen sowie erhöhter Mortalität und wird wiederum als bedeutender pathogenetischer Faktor für das Auftreten des geriatrischen Frailty-Syndroms angesehen. Polypharmazie ist aber auch ein Risikofaktor für Überversorgung mit inadäquaten Medikamenten. Die Reduktion von Polypharmazie (*Deprescribing*) stellt somit eine vielversprechende Intervention zur Verbesserung bzw. Stabilisierung des gesundheitlichen Zustands von Frailty-Patienten dar.

Die Versorgung von Patienten mit Frailty-Syndrom im ambulanten Sektor gestaltet sich aufgrund der Komplexität der Probleme oft als schwierig. Fehlende Koordination der Versorgung fördert wiederum ungeplante Krankenhausaufnahmen in Folge von Sturzereignissen, Schmerzsyndromen, Verwirrtheits- oder Schwächezuständen. Da Klinikaufenthalte bei diesen vulnerablen Patienten kostenintensiv sind und zudem wiederum selbst ein hohes Risiko darstellen, ist die Senkung der Hospitalisierungsrate als ein wichtiges Ziel des hausärztlichen Case Managements beim geriatrischen Frailty Syndrom anzusehen. Dieses soll auf dem Wege einer Stärkung des Empowerments von Patienten und ihren Angehörigen erreicht werden. Die Herausforderung von Patienten mit Frailty-Syndrom besteht für Hausärzte nicht nur in einer besseren Organisation der Primärversorgung, sondern vor

allem darin, gemeinsam mit dem Patienten, den pflegenden Angehörigen und ggf. dem Pflegedienst eine Priorisierung möglicher therapeutischer Maßnahmen anhand der individuellen Präferenzen des Patienten vorzunehmen. Daran anknüpfend, sollen Familienkonferenzen als ein möglicherweise wirkungsvolles Instrument für die Versorgung der Patienten mit Frailty-Syndrom in der Hausarztpraxis in der Studie zum Einsatz kommen.

1.4 Gegenstand und Zielstellung der Studie

Die Studie untersucht die Effektivität von Familienkonferenzen (Patient, Hausarzt, Angehöriger, Pflegedienst) im Rahmen einer komplexen Intervention im Hinblick auf patientenbezogene Outcomes bei älteren Patienten mit geriatrischem Frailty-Syndrom und Polypharmazie. Es wir hierfür zum einen geprüft, ob die Intervention im Vergleich zur Kontrolle zu einer Verringerung der durchschnittlichen Anzahl der Klinikaufenthalte pro Patient (Hospitalisierungsrate) führt.

Zum anderen wird untersucht, ob die Intervention im Vergleich zur Kontrolle:

1) zu einer Verbesserung des gesundheitlichen Zustands der Patienten hinsichtlich Ausprägung des Frailty-Syndroms, Kognition, Lebensqualität, Aktivitäten des täglichen Lebens,

2) zu einer Verringerung der Verschreibungshäufigkeit von potenziell inadäquaten Medikamenten (PIM),

3) zu einer besseren Berücksichtigung der individuellen Präferenzen der Patienten sowie der vorhandenen familiären Ressourcen aus Sicht von Patienten, Angehörigen und Hausärzten führt und

4) sich als kosteneffektiv hinsichtlich der Erreichung der o.g. Ziele erweist.

Die Untersuchung erfolgt als pragmatische cluster-randomisierte kontrollierte Studie (cRCT).

1.5 vor Ort an der Studie weitere beteiligte Institute/Kliniken/Praxen (Anschrift, Telefon)
keine

1.6 Wie wird die Studie finanziert? Kostenträger? Sponsor?
Innovationsausschuss beim Gemeinsamen Bundesausschuss
Förderkennzeichen beim Innovationsausschuss: 01VSF17053
Konsortialführung: Institut für Allgemeinmedizin, Universitätsklinikum Düsseldorf

1.7 Handelt es sich um eine multizentrische Studie?
Ja
Die Rekrutierung und Datenerhebung erfolgt an zwei Standorten (Düsseldorf und Rostock).
Die Prozessevaluation wird vom Institut für Gesundheits- und Pflegewissenschaft der Medizinischen Fakultät der Martin-Luther-Universität Halle-Wittenberg verantwortet (Ausführungen zur Prozessevaluation finden sich unter 2.4).

1.8 Voten anderer Ethikkommissionen
Wurde bereits bei einer anderen Ethikkommission in Deutschland ein Antrag gleichen Inhalts

gestellt?
Nein

1.9 Welche speziellen Gesetze/Verordnungen müssen Sie bei Ihrer Studie beachten?
Bundesdatenschutzgesetz (Fassung des BDSG vom 25.05.2018 nach EU-Richtlinien) und Datenschutzgrundverordnung (DSGVO)

1.10 Angaben zu wirtschaftlichen und anderen konfligierenden Interessen der Studienleitung im Zusammenhang mit der Studie
Es bestehen keine Interessenskonflikte.

2. Kurzcharakterisierung der Studie

2.1 Art der Prüfung
Sonstige, und zwar: Prüfung der Effektivität einer komplexen Intervention

2.2 Studiendesign
pragmatische cluster-randomisierte kontrollierte Studie (cRCT) mit komplexer Intervention (siehe Abbildung 1: Studienverlauf).

2.2.1 Methoden
Es handelt sich um eine cluster-randomisierte kontrollierte Interventionsstudie. Die Cluster-Randomisierung erfolgt auf Ebene von Hausarztpraxen.

Rekrutierung und Randomisierung
Rekrutiert werden insgesamt 136 Hausärzte in Rostock (n=46) und Düsseldorf (n=90). Die anschließende Rekrutierung der insgesamt 676 Patienten erfolgt anhand der festgelegten Ein- und Ausschlusskriterien durch die Hausärzte in den Praxen (Rostock n= 228, Düsseldorf n= 448). Nach der Baseline-Erhebung erfolgt die Randomisierung der Hausärzte in zwei Arme (Intervention und Kontrolle). Das komplexe Design der Intervention erlaubt keine Verblindung.

Kontrolle
In den zur Kontrollgruppe zählenden Hausärzten findet keine der nachfolgend aufgeführten Interventionsmaßnahmen statt, stattdessen wird die Regelversorgung (care as usual) beobachtet sowie Fortbildungen zum Thema Geriatrie angeboten.

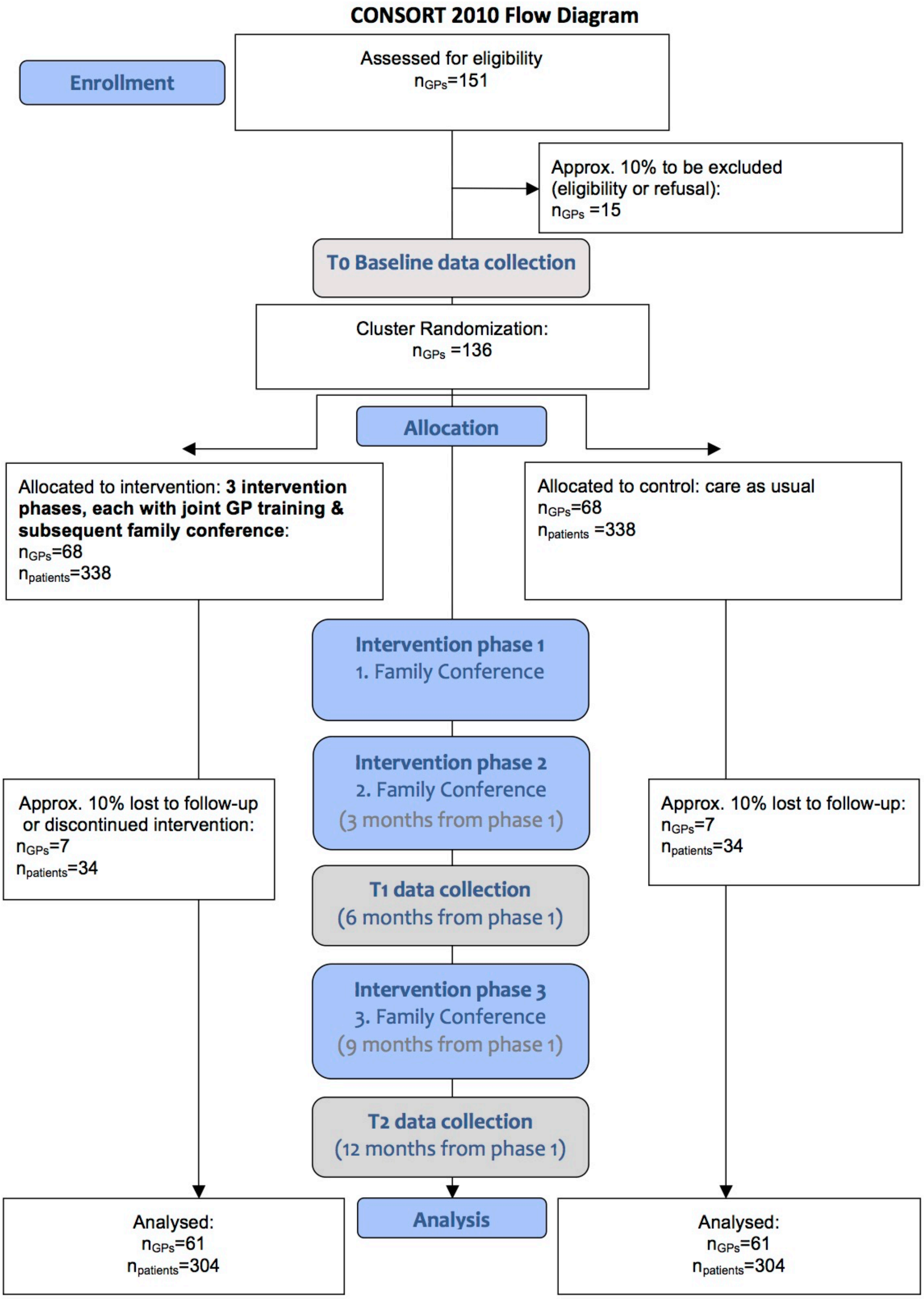

Abbildung 1 – Studienverlauf

Intervention
Die Intervention erfolgt in zwei Phasen:

Phase 1 (Vorbereitung der Intervention): Die teilnehmenden Hausärzte der Interventionsgruppe erhalten drei inhaltlich aufeinander aufbauende Fortbildungen, bei denen fachliche Kenntnisse sowie mittels Kommunikationstraining die Kompetenzen für die Durchführung strukturierter Familienkonferenzen vermittelt werden (2 verpflichtende Präsenzveranstaltungen, 1 fakultative Fortbildung ggf. als *Webinar*). In diesem Rahmen werden den Ärzten evidenzbasierte Fachinformationen sowie schriftliche Materialien zur Unterstützung des Gesprächs mit Patienten und Angehörigen zur Verfügung gestellt, die im Sinne einer Toolbox bedarfsadaptiert in die Familienkonferenz einfließen sollen. Obligater Inhalt ist ein Medikations-Check mit gemeinsamer Priorisierung der Arzneimitteltherapie im Rahmen der Familienkonferenz. Die Hausärzte können über eine Hotline fakultativ ein individuelles Medikations-Review durch einen klinischen Pharmakologen anfordern. Weitere fakultative Elemente der Toolbox sind:

a) Beratung über Maßnahmen zu Mobilitätssicherung, Sturzprävention und Schmerztherapie;
b) Gemeinsame Erstellung eines Notfallplans, ggf. unter Berücksichtigung einer Patientenverfügung;
c) Gespräch über evtl. bestehenden pflegerischen und psychosozialen Unterstützungsbedarf, Vermittlung einer Pflegeberatung durch einen lokalen Pflegestützpunkt.

Phase 2 (Durchführung der Intervention in den Hausarztpraxen): Durchführung von drei Familienkonferenzen pro Patient zu Beginn der Studie sowie nach 3 und 9 Monaten für jeweils 30-45 Minuten im Rahmen eines Hausbesuchs unter Einbeziehung der pflegenden Angehörigen und/oder Pflegedienste. Im gemeinsamen Gespräch werden:
a) zunächst die allgemeinen Präferenzen des Patienten hinsichtlich seiner gesundheitlichen Versorgung herausgearbeitet (z.B. zu möglichen Indikationen für eine stationäre Einweisung),
b) ein Medikations-Check erstellt, bei dem die initial erarbeiteten Präferenzen angewendet werden,
c) je nach Bedarf weitere Themen aus der o.g. Toolbox bearbeitet.

Im Anschluss erfolgt eine Zusammenfassung mit Dokumentation durch den Hausarzt sowie eine Vereinbarung über das weitere Procedere wie z.B. die Folgetermine.
Die Laufzeit pro Patient beträgt 12 Monate mit Datenerhebungen zu T0 (Baseline), T1 (6 Monate) und T2 (12 Monate).

Datenerhebung
Bei allen Patienten (beider Studienarme) werden vor Beginn (Baseline), während (6 Monate) und am Ende der Intervention (12 Monate) verschiedene Parameter erhoben bzw. Erhebungsinstrumente eingesetzt:
Basisdaten (einmalig vor Beginn und ggf. im Verlauf)
1. Alter
2. Geschlecht
3. Schulabschluss
4. Familienstand
5. Wohnsituation

6. Pflegegrad
7. Grad der Behinderung

Vor Beginn, während und am Ende der Intervention:

- durchschnittliche Anzahl und Dauer der Klinikaufenthalte pro Patient (zur Bestimmung der Hospitalisierungsrate)
- Relevante Diagnosen
- Gewichtsverlauf
- körperliche Schwäche (Hand-Greifkraft mit Dynamometer)
- subjektive Einschätzung des Erschöpfungsgrades
- Gehgeschwindigkeit
- Grad der körperlichen Aktivität
- Kognition (Episodic memory, Executive function → CERAD-Test)
- Anzahl eingenommene Medikamente/Wirkstoffe u.a. zur Bestimmung potentiell inadäquater Medikation
- Psychologischer Status (Geriatric Depression Scale (GDS))
- gesundheitsbezogende Lebensqualität (EQ-5D, VAS-Skala)
- Sturzhäufigkeit
- Activities in daily living (Barthel-Index)
- Blutdruck
- Puls
- Kardiovaskuläre Morbidität (Herzinfarkt, Apoplex)
- Mortalität
- Kostenaspekte (*incremental cost-effectiveness ratio*)

2.3 Studienteilnehmerinnen/-teilnehmer

Das Rekrutierungsverfahren ist mehrstufig. Nacheinander werden erst Hausärzte dann Patienten rekrutiert. Insgesamt sollen 676 Patienten durch 136 Hausärzte (5 Patienten pro Hausarzt) eingeschlossen werden.
Der Patient wird bei der Rekrutierung durch den Hausarzt gebeten, einen Angehörigen als Hauptbezugsperson zu benennen, welcher bei den Familienkonferenzen dabei sein soll. Ggf. wird zudem ein Vertreter des ambulanten Pflegedienstes zur Teilnahme eingeladen.

2.3.2 Ein- und Ausschlusskriterien:

Hausärzte: können teilnehmen, wenn sie als Fachärzte für Allgemeinmedizin- oder hausärztlich tätige Fachärzte für Innere Medizin bei der zuständigen Kassenärztlichen Vereinigung registriert sind.
Patienten: Der Einschluss erfolgt, wenn alle folgenden Kriterien erfüllt sind:

1) Positives Screening auf das geriatrische Frailty-Syndrom
2) Alter 70 Jahre
3) regelmäßige Einnahme von 5 Wirkstoffen (Polypharmazie)
4) Pflegebedürftigkeit (Pflegegrad 1 oder höher)
5) Pflegerische Versorgung in eigener Wohnung durch Familienangehörige und/oder ambulanten Pflegedienst

Ein Ausschluss erfolgt, wenn nach Einschätzung des Hausarztes ein oder mehrere der folgenden Kriterien vorliegen:

1) Mittelschwere oder schwere Demenz
2) reduzierte Lebenserwartung von 6 Monaten (Palliativversorgung)

3) Versorgung in vollstationärer Pflegeeinrichtung
4) keine ausreichenden Sprachkenntnisse in Deutsch von Patient und pflegenden Angehörigen oder Nicht-Verfügbarkeit eines Dolmetschers

Angehörige bzw. Vertrauenspersonen und Pflegedienst: können teilnehmen, wenn sie vom Patienten festgelegt werden. Dieser entscheidet auch, ob ggf. ein Vertreter des Pflegedienstes an den Familienkonferenzen beteiligt werden soll.

Begründen Sie bitte, inwieweit die gewählte Geschlechterverteilung zur Feststellung möglicher geschlechtsspezifischer Unterschiede (z.B. in der Wirksamkeit der Behandlung, der Unbedenklichkeit des Medizinproduktes) angemessen ist.
Patientinnen wie Patienten werden konsekutiv in die Studie eingeschlossen ohne Rücksicht auf das Verhältnis der Geschlechter.
Bei der Auswertung wird das Geschlecht durchgehend als einer der relevanten Faktoren, der die Ergebnisse beeinflussen kann, berücksichtigt. Insbesondere bei Fragen des Outcomes der Versorgung werden mögliche Unterschiede für Männer und Frauen explizit beschrieben.

2.4 Prozessevaluation:
Um den Veränderungsprozess nachvollziehen zu können und zur Verallgemeinerbarkeit der Studienergebnisse ist eine umfassende Erhebung von Prozessdaten begleitend zur pragmatischen Interventionsstudie unerlässlich. Die Prozessevaluation erfolgt entsprechend internationaler Empfehlungen für die Prozessevaluationen von komplexen Interventionen zu den Themen: Implementierung, Wirkmechanismen und Kontext. Es stehen folgende Fragen im Mittelpunkt: Was ist implementiert und wie ist es implementiert (*fidelity, dose, adapation, reach*)? Wie führt die verabreichte Intervention zu Veränderungen (*particpant response, mediators, unanticipated pathways and consequences*)? Wie beeinflusst der Kontext die Implementierung, die Mechanismen und die Ergebnisse?
Daher sollen unterschiedliche Prozessparameter auf Cluster- und Individualebene mit qualitativen und quantitativen Methoden erhoben werden (vgl. Tab. 1). Zur Untersuchung der Machbarkeit und zur Optimierung der Intervention werden nach der Pilotierung der Familienkonferenzen halbstandardisierte Interviews mit den Hausärztinnen bzw. Hausärzten und kurze Telefoninterviews mit den Patientinnen bzw. Patienten durchgeführt. Der Rekrutierungsprozess, einschließlich der Gründe zur Nichtteilnahme, wird sowohl auf Cluster- als auch Individualebene dokumentiert. Kontextfaktoren werden unter Berücksichtigung wichtiger struktur- und prozessbedingter Aspekte (z. B. sozioökonomischer und soziodemografischer Merkmale der Hausärzte und Patienten) zu Studienbeginn erhoben.
Zur Bestimmung der *intervention fidelity* werden sowohl von den Schulenden als auch von den Hausärzten nach jeder Fortbildung ein strukturierter Dokumentationsbogen ausgefüllt. Zusätzlich werden die Familienkonferenzen anhand eines halbstandardisierten Fragebogens zu t_0, nach drei und nach sechs Monaten evaluiert, der von den Hausärzten unmittelbar im Anschluss an die Familienkonferenz ausgefüllt werden soll.
Die Einstellungen bzgl. der COFRAIL-Intervention und die Erfahrungen, die die Hausärzte damit gemacht haben, sollen im Rahmen von vier Fokusgruppen mit einer Gelegenheitsstichprobe von sechs bis zwölf Teilnehmer/innen am Studienende diskutiert werden. Zudem werden mit einer Zufallsstichprobe von etwa 10 Hausärzten pro Region halbstandardisierte Einzelinterviews durchgeführt.
In einer Substichprobe von 10 % werden die Erfahrungen der Familien (z. B. Berücksichtigung von Präferenzen; Veränderungen in der Arzt-Patient-Kommunikation; Barrieren und

Förderfaktoren) nach zwölf Monaten anhand von halbstandardisierten Einzelinterviews erhoben.
Die Prozessevaluation ist rein explorativ. Alle quantitativen Daten werden deskriptiv analysiert, alle qualitativen Daten anhand der qualitativen Inhaltsanalyse nach Mayring. (Alle Dokumente zur Prozessevaluation hinsichtlich der Einwilligung in diese finden sich in der Anlage 5-7.)

Tabelle 1: Inhalte der Prozessevaluation.

**Fokus**	**Erhebungsmethode/Dokumentation**	**Erhebungszeitpunkt**
Machbarkeit der Intervention	Pilotierung der Familienkonferenzen mit 2 Hausärztinnen bzw. Hausärzte/Region: **halbstandardisiertes Interview** mit Hausärzten**; kurzes Telefon Interview** mit Patientinnen bzw. Patienten und Angehörigen	Pilotierung, vor to
Rekrutierungsverlauf der Hausärzte und Patienten	**Protokoll**/Region	t_0
Gründe für nicht-Teilnahme oder vorzeitiges Beenden der Studienlaufzeit	**Strukturierte Abfrage und Dokumentation** der Gründe	t_0-t_2
Beschreibung wesentlicher Struktur- und Prozess-bezogener Faktoren auf Cluster- und Patientenebene	**CRF-Baseline Daten**/Cluster und Patientinnen bzw. Patienten	t_0
Übertragung der Intervention (*intervention fidelity*)	Obligatorische Fortbildungseinheiten: **Strukturiertes Protokoll** für jede Fortbildungseinheit<br><br>Inanspruchnahme der fakultativen Fortbildungseinheit: **Standardisierte Dokumentation**	t_0 (unmittelbar im Anschluss der Fortbildungen)
Auswirkungen der Schulungsintervention	Alle Teilnehmenden des Fortbildungsprogramms: **Standardisierter Fragebogen** → Evaluation des Programms<br>• Einstellungen<br>• Akzeptanz<br>• Selbstwirksamkeit<br>• Erwartungen	t_0 (vor und nach der Fortbildungsveranstaltung)
Anwendung der Schulungsinhalte	Alle Hausärztinnen bzw. Hausärzte:<br>**Halbstrukturierte Protokolle** → Evaluation der Familienkonferenzen<br>• Akzeptanz<br>• Inhalte<br>• Dauer<br>• Umsetzbarkeit<br>• Anpassungsbedarf	t_0, nach 3 und 6 Monaten (unmittelbar nach den Familienkonferenzen)
Erfahrungen der Hausärztinnen und Hausärzte (z. B. Einstellung bzgl. der Intervention; Veränderungen der Arzt-Patient-Kommunikation; Barrieren und Förderfaktoren)	**4 Fokusgruppen**; 2/Region mit 6 bis 12 Hausärztinnen bzw. Hausärzte<br><br>**Halbstandardisierte Interviews**; Zufallsstichprobe von 10 Hausärztinnen bzw. Hausärzte /Region	t_2
Erfahrungen der Patientinnen und Patienten (z. B. Berücksichtigung von Präferenzen; Veränderungen der	**Halbstandardisierte Interviews**; 10 % der Teilnehmenden/Region	t_2

Arzt-Patient-Kommunikation; Barrieren und Förderfaktoren)		

3. Schutz und Sicherheit der Studienteilnehmerinnen und -teilnehmer

3.1 und 3.2 Beschreibung des Verfahrens zur Rekrutierung von Studienteilnehmerinnen und -teilnehmern und zur Erlangung der informierten Einwilligung zur Studienteilnahme der Studienteilnehmerinnen/-teilnehmer, ggf. der Eltern oder des gesetzlichen Vertreters

Rekrutierungsverfahren:
Das Rekrutierungsverfahren ist mehrstufig. An zwei Standorten (Düsseldorf, Rostock) werden Hausärzte solange in Wellen angeschrieben bis die notwendige Anzahl erreicht ist. Dazu werden Hausärzte postalisch zur Teilnahme an einer Fortbildung bzw. zur Studie eingeladen. Im nächsten Schritt werden alle interessierten Hausärzte über die Studie mündlich oder schriftlich informiert. Nach Interessensbekundung wird die unterschriebene Einwilligungserklärung von den Hausärzten eingeholt.
Im Anschluss erstellt der Hausarzt anhand der Abrechnungsziffer für geriatrische Erkrankungen im letzten Quartal (EBM Ziffer 03362) eine Liste mit grundsätzlich in Frage kommenden Patienten. Der Hausarzt und die im Projekt eingestellte Studienassistenz prüfen gemeinsam anhand der Ein- und Ausschlusskriterien, welche Patienten potentiell für eine Studienteilnahme geeignet sind (siehe 2.3.2). Nachfolgend werden solange Patienten konsekutiv durch den Hausarzt kontaktiert und deren Teilnahmebereitschaft erfragt, bis die Anzahl von fünf Patienten pro Hausarzt erreicht oder die Liste abgearbeitet ist. Die schriftliche Einwilligung der Patienten wird durch die Hausärzte eingeholt (Einwilligungserklärung), danach erfolgt die Weitergabe der Kontaktdaten an das jeweilige Studienzentrum. Nach der Erhebung der Baseline-Daten (t0) erfolgt dann die Randomisierung in Kontroll- und Interventionsgruppe. Die Patienten der Interventionsgruppe werden gebeten, eine Bezugsperson/ einen Angehörigen und ggf. einen Vertreter des Pflegedienstes zu benennen. Die weiteren Beteiligten werden ebenfalls über die Studie informiert.
Alle Rekrutierungsunterlagen für die Patienten und Hausärzte finden Sie in der Anlage zu diesem Antrag. Diese beinhalten An- und Infoschreiben für Hausarzt und Patient, Checkliste zum Einschluss der Patienten bzw. Grafik zum Studienablauf (Hausarzt) und Einwilligungserklärung (Patient und Hausarzt). Ferner sind die Infoschreiben für Bezugspersonen/ Angehörige und Vertreter des Pflegedienstes beigefügt (siehe Anlage 1-4).

Aufklärung und Einwilligung:
Die Hausärzte werden durch das jeweilige Studienzentrum schriftlich und mündlich über die Studie informiert und willigen schriftlich ein. Die Patienten werden zunächst durch die Hausärzte, dann ggf. durch die Studienmitarbeiter über die Studie und Datenschutzbestimmungen ausführlich informiert und aufgeklärt. Eine Studienteilnahme und die damit verbundene Datenerhebung sind erst nach Einwilligung der Patienten möglich. Alle Patienteninformationsmaterialien (Anschreiben, Informationsschreiben und Einwilligungserklärungen für Patienten) sowie Arztinformationen sind diesem Antrag beigefügt (siehe Anlage 1 und 2).

Dauer der Rekrutierung (Materialien wie Anzeigen, Flyer etc. beilegen)
Die Rekrutierung der Hausärzte erfolgt nach Vorlage des Ethikvotums ab 01.12.2018 und ist

innerhalb von 6 Monaten abgeschlossen. Ab 01.04.2019 erfolgt die Rekrutierung der Patienten durch die Hausärzte innerhalb von 4 Monaten.

3.3 Besonders schutzbedürftige Personen:
Nehmen an der Studie Personen unter 18 Jahren teil? Nein
Nehmen nicht-einwilligungsfähige Erwachsene teil? Nein

3.4 Begründung für den Einschluss von besonders schutzbedürftigen Personen
(wie z.B. gesunde bzw. kranke Minderjährige, nichteinwilligungsfähige Erwachsene) Warum kann die Studie nicht an einwilligungsfähigen Erwachsenen durchgeführt werden?
Trifft nicht zu

3.5 Von wem werden die Studienteilnehmerinnen/ -teilnehmer vor, während und nach der Studie ärztlich betreut?
(z.B. Abstimmung mit dem Hausarzt /oder Hausärztin? Kontrolle anderer Medikationen? Kontrolle von Blutspiegeln?)
Die teilnehmenden Patienten werden vor, während und nach der Studie wie gewohnt von ihrem Hausarzt betreut.
Bei Rücknahme der Einwilligung auf Seiten der Patienten ist deren Behandlung im Rahmen der Regelversorgung beim Hausarzt und ggf. ambulanter Pflege unverändert gesichert.

3.6 Erhalten die teilnehmenden Personen eine Aufwandsentschädigung bzw. eine Bezahlung?
Alle teilnehmenden Ärzte erhalten unabhängig von ihrer Gruppenzuordnung (Intervention / Kontrolle) pro Patient 200€ Aufwandsentschädigung. Die Patienten und deren Vertrauensperson bzw. Vertreter vom ambulanten Pflegedienst erhalten keine Vergütung für die Teilnahme.

3.7 Ist zugunsten der Studienteilnehmerinnen/ -teilnehmer eine Versicherung (evt. auch Wegeversicherung) abgeschlossen worden?
Nein

4. Dokumentation, Auswertung, Berichterstattung

4.1 Welche Art der Dokumentation ist vorgesehen? (Dokumentationsbögen ggf. beifügen)
Die Zielvariable der primären Forschungshypothese ist die Verringerung der durchschnittlichen Anzahl der Krankenhausaufenthalte pro Patient (Hospitalisierungsrate) innerhalb von 12 Monaten nach Interventionsbeginn.
Die sekundäre Forschungshypothese befasst sich mit der Verbesserung des gesundheitlichen Zustandes des Patienten hinsichtlich der Ausprägung des Frailty-Syndroms (Gewichtsverlauf, körperliche Schwäche, Erschöpfungsgrad, Gehgeschwindigkeit, körperliche Aktivität), der Kognition, der Zahl der eingenommenen Medikamente, dem psychologischem Status, der gesundheitsbezogenen Lebensqualität, der Sturzhäufigkeit, den Aktivitäten des täglichen Lebens und den Kosten.
Zusätzlich werden relevante Diagnosen sowie die Sicherheitsparameter Blutdruck, Puls, kardiovaskuläre Morbidität und Prognose bezogen auf die Mortalität erfasst.
Die nachfolgende Tabelle beschreibt wo und wie die Zielgrößen erhoben werden.

Tabelle 2: Übersicht der Datenerhebungen und -quellen

Research hypothesis	Item	Data source
Primary	Number/duration of hospital stays	GP data collection form; Patient data collection form
Secondary	Relevant diagnoses	GP data collection form
Secondary	Number of drugs taken, PZN	GP data collection form; Patient data collection form
Secondary / safety parameter	Blood pressure	GP data collection form
Secondary / safety parameter	Heart rate	GP data collection form
Secondary / safety parameter	Cardiovascular morbidity (myocardial infarction, apoplexy)	GP data collection form
Secondary / safety parameter	Prognosis/ mortality	GP data collection form
Secondary	Weight trend	Patient data collection form
Secondary	Physical weakness	Patient data collection form
Secondary	Degree of exhaustion	Patient data collection form
Secondary	Walking speed	Patient data collection form
Secondary	Degree of physical activity	Patient data collection form
Secondary	Cognition	Patient data collection form
Secondary	Psychological status	Patient data collection form
Secondary	Health-related quality of life	Patient data collection form
Secondary	Frequency of falls	Patient data collection form
Secondary	Activities of daily living	Patient data collection form

Secondary	Costs	Patient data collection form

Datenfluss:
Die Erhebung der Baseline-Daten sowie die Eingabe der Daten in eine Datenbank (ORACLE-Datenbank, siehe auch Punkt 4.4) erfolgt durch die jeweilige Studienassistenz der Studienzentren (t0).
Sechs Monate bzw. 12 Monate nach Erhebung der Baseline-Daten erfolgt durch diese und den Hausarzt die Erhebung der Follow-up-Daten (t1, t2) sowie die Eingabe der Daten in die Datenbank.

4.2 Ist ein externer Monitor vorgesehen?
Nein

4.3. Ist die Mitarbeit einer Statistikerin/ eines Statistikers vorgesehen?
Ja: Dipl.-Math. Birgitt Wiese, Institut für Allgemeinmedizin, Medizinische Hochschule Hannover

4.4 Vorgesehene Maßnahmen zum Schutze der erhobenen Daten
Die Daten werden mittels eines webbasierten Dateneingabesystems secuTrial® lokal in den Zentren (Düsseldorf und Rostock) in eine zentrale ORACLE-Datenbank (Oracle 9i) eingegeben. Der Zugang zur internen Datenbank und dem Webserver wird durch zwei hintereinander geschaltete Firewalls kontrolliert. Der Datentransfer geschieht durch eine 128 bit SSL Verschlüsselung. Es erfolgt keine lokale Datenspeicherung. secuTrial® erfüllt die Anforderungen der FDA (21 CFR Part 11) und entspricht den Richtlinien für Good Clinical Practice (GCP).
Die Daten werden mit einem Pseudonym ohne Identifikationsdaten gespeichert. Die Mitglieder der Studiengruppe haben gemäß eines detaillierten Rollen- und Rechtekonzepts Zugang zum elektronischen Dateneingabesystem. Ein Audit Trail gewährleistet ein automatisches Protokoll aller Dateneingaben, -änderungen und -löschungen. Verschiedene Maßnahmen zur Datenqualitätssicherung werden implementiert: Dazu gehören Online-Plausibilitätschecks während der Dateneingabe, Datenkontrollen nach Dateneingabe sowie Nutzerschulungen vor Erfassung der Daten. Zudem werden alle an Datenerhebung und -eingabe Beteiligten durch das Datenmanagement geschult (Studienassistenten, Studenten) und erhalten ein Handbuch, welches alle Funktionen beschreibt und erklärt.

4.5 Sind Abbruchkriterien festgelegt worden?
- für die einzelnen **Studienteilnehmerinnen / -teilnehmer**: Ja
    - Die Teilnahme kann von den einzelnen Patienten jederzeit ohne Angabe von Gründen zurückgezogen werden.
- für die Beendigung der **gesamten Studie**: Ja
    - Als Kriterium für den Abbruch der Studie wird der Anstieg der Hospitalisierungsrate von > 30% in Kontroll- und/oder Interventionsgruppe definiert.

5. Abwägung des Schaden/Nutzenpotentials

5.1 Schaden und Risiken

Welche Arten sind die möglichen Risiken, Beeinträchtigungen, Belastungen oder sonstige Nachteile für die an der Studie teilnehmenden Personen?

Die Intervention zielt auf eine Verbesserung der Kommunikation zwischen Hausärzten, Patienten und Angehörigen. Medizinische Risiken sind somit nicht zu erwarten, da in der Studie auf die medizinische und pharmakologische Behandlung keinen unmittelbaren Einfluss genommen wird.

Es wird angenommen, dass für die an der Studie teilnehmenden Patienten kein Risiko besteht oder Schäden zu erwarten sind. Dennoch werden als Sicherheitsparameter neben der Hospitalisierungsrate der Blutdruck, der Puls und die Mortalität definiert, welche von der Projektleitung und von den Kooperationspartnern regelmäßig analysiert werden.

Patienten, sowohl in Interventions- als auch in Kontrollgruppe werden durch speziell geschulte Studienassistenten befragt. Dies geschieht nur mit der ausdrücklichen Einwilligung der Patienten. Zudem werden die Studienassistenten vom Studienteam so eingearbeitet, dass sie für den Umgang mit älteren Menschen sensibilisiert sind. Den Patienten wird ein ausreichender zeitlicher Rahmen für die Beantwortung der Fragen bzw. Durchführung der Tests gegeben, da diese als anstrengend empfunden werden können. Pausen/Unterbrechungen der Befragungen werden bei Bedarf angeboten.

5.2 Inwieweit weichen ggf. Studienbezogene Maßnahmen von der üblichen Routinebehandlung ab?

Alle Patienten erfahren die übliche Routinebehandlung im Rahmen der normalen Versorgung durch ihre beteiligten Ärzte. Die pflegerische Leistung erfolgt weiter wie bisher durch den ambulanten Pflegedienst.

In der Interventionsgruppe bieten wir zusätzlich:

Durchführung von drei Familienkonferenzen pro Patient zu Beginn der Studie sowie nach 3 und 9 Monaten für jeweils 30-45 Minuten im Rahmen eines Hausbesuchs unter Einbeziehung der pflegenden Angehörigen und/oder Pflegedienste. Im gemeinsamen Gespräch werden:

- zunächst die allgemeinen Präferenzen des Patienten hinsichtlich seiner gesundheitlichen Versorgung herausgearbeitet (z.B. zu möglichen Indikationen für eine stationäre Einweisung),
- ein Medikations-Check erstellt, bei dem die initial erarbeiteten Präferenzen angewendet werden,
- je nach Bedarf weitere Themen aus der Toolbox (nicht-pharmakologische Interventionen zu Mobilitätssicherung, Notfallplanung, Bereitstellung psychosozialer Hilfen) bearbeitet und eine Zusammenfassung durch den Hausarzt über das weitere Procedere erstellt

(siehe hierzu unter Punkt 2.2.1. Intervention)

5.3 Nutzen und Chancen

Welcher Art ist der zu erwartende Nutzen?

Eigennutzen: Durch die Verbesserung der Arzneimitteltherapiesicherheit (AMTS) mit Reduktion der Zahl verordneter potenziell inadäquater Medikamente (PIM) sowie mit Reduktion arzneimittelbedingter Nebenwirkungen (Sturzrate, Hospitalisierungen) wird die Patientensicherheit erhöht. Zudem erfolgt eine relative Verbesserung des Gesundheitszustandes im Vergleich zur Kontrollgruppe, durch eine Verringerung der Ausprägung des Frailty-Syndroms im Verlauf. Darüber hinaus werden die Partizipation und die Selbstbestimmung von Patienten mit Frailty-Syndrom durch die Stärkung der

gemeinsamen Priorisierung und partizipativen Entscheidungsfindung durch die Teilnahme an der Familienkonferenz verbessert.
Gruppennutzen: Ziel der Familienkonferenz ist die Verbesserung der kommunikativen Kompetenzen von Ärzten im Umgang mit komplexen Behandlungsproblemen. Durch diese Methode wird die Kommunikation und Kooperation zwischen den beteiligten Akteuren verbessert. Somit wird eine strukturiertere Verständigung über Probleme sowie das gezielte Suchen nach Lösungen ermöglicht. Davon profitieren auch die Patienten, die nicht direkt an der Studie teilnehmen.
Fremdnutzen: Die gewonnenen Erkenntnisse können dazu genutzt werden, um den Einsatz des Instrumentes *Familienkonferenz* auch für andere komplexe Versorgungsbereiche zu diskutieren. Bei positiver Evaluation können diese Ergebnisse in die Entwicklung didaktischer Formate für die ärztliche Aus-, Weiter-, und Fortbildung einfließen.

5.4 Begründen Sie bitte, warum der mögliche Schaden im Verhältnis zu dem zu erwartenden Nutzen Ihrer Ansicht nach vertretbar ist.
Vor dem Hintergrund der Verbesserung der Patientensicherheit ist ein Schaden nahezu ausgeschlossen. Die Teilnahme der Patienten ist freiwillig und die Einwilligung kann jederzeit zurückgezogen werden, ohne dass Nachteile entstehen. Durch die Verknüpfung der Kompetenzen der den Patienten betreuenden Berufsgruppen kann im Falle eines auftretenden Problems eine effizientere Lösung gewährleistet werden.

6. Unterschriften und Erklärung

6.1 Stellungnahme und Unterschrift der Direktorin/ des Direktors der Klinik bzw. des Institutes (mit Stempel)
Mit der Durchführung der unter 1.1 genannten Studie bin ich einverstanden. Ich bestätige, dass die Ressourcen (Anzahl und Qualifikation der Mitarbeiter, Infrastruktur, Geräte und Räumlichkeiten) für eine erfolgreiche Durchführung vorhanden sind.
Außerdem bestätige ich, dass die Rekrutierung der Studienteilnehmerinnen / -teilnehmer nicht durch konkurrierende Studien gefährdet wird.

(Stempel) Rostock, den
Unterschrift (Prof. Dr. Attila Altiner)

Anlagen:
Anlage 1 – Rekrutierungsunterlagen Hausärzte (Anschreiben, Studieninformationen, Faxantwort, Einwilligungserklärung)
Anlage 2 – Rekrutierungsunterlagen Patienten (Anschreiben, Studieninformationen, Einwilligungserklärungen)
Anlage 3 – Studieninformationen an Bezugsperson/ Angehörige,
Anlage 4 – Studieninformationen an Pflegedienst
Anlage 5 – Dokumente Prozessevaluation Hausärzte
Anlage 6 – Dokumente Prozessevaluation Patienten
Anlage 7 – Dokumente Prozessevaluation Bezugsperson/ Angehörige

COFRAIL - Statistischer Analyseplan

1. Einleitung

Die COFRAIL-Studie ist eine multizentrische cluster-randomisierte kontrollierte Interventionsstudie zur Erhöhung der Patientensicherheit durch den Einsatz von Familienkonferenzen in der hausärztlichen Versorgung.

2. Datengrundlage

Es werden 676 Patienten in 138 allgemeinmedizinischen Praxen rekrutiert werden, wobei die Praxen in die Interventions- bzw. die Kontrollgruppe im Verhältnis 1:1 randomisiert werden, sodass entsprechend 338 Patienten der Interventionsgruppe und 338 Patienten der Kontrollgruppe zugeordnet werden. Es werden Daten zu Baseline T0, zur Zwischenuntersuchung T1 (nach 6 Monaten) und zur Abschlussuntersuchung T2 (nach 12 Monaten) erhoben. Zusätzlich werden in der Interventionsgruppe Daten zu drei Familienkonferenzen, die zwischen T0 und T1 bzw. zwischen T1 und T2 liegen, erfasst. Dabei werden soziodemografischen Daten, Daten zu Lebensqualität (EQ-5D-5L), Gedächtnistests (CERAD), Körperliche Funktionalität, Geriatrische Depressionsskala, die Medikation, Krankenhausaufenthalte, Arztkontakte, Sturzanamnese, sowie weitere gesundheitsökonomisch relevante Daten erhoben. Die Daten werden im webbasierten EDC-System secuTrial® in eine zentrale ORACLE-Datenbank erfasst. Um eine hohe Datenqualität zu erreichen sind umfangreiche Plausibilitätsprüfungen im Eingabesystem implementiert, zusätzlich werden nach Eingabe der Daten weitere Datenkontrollen durchgeführt.

3. Ziel, Endpunkte und statistische Analysen

Das Hauptzielkriterium - der primäre Endpunkt – ist die Anzahl der Hospitalisierungen pro Patient innerhalb der Beobachtungszeit von 12 Monaten.

Die sekundären Endpunkte sind die folgenden:

- Medikation: Anzahl Medikamente pro Patient, Drug Burden Index (DBI) definiert als Anzahl anticholinerger oder sedativer Medikationen sowie die Prävalenz von potentiell inadäquater Medikation
- Handkraftmessung
- Kognition: Punktwerte der CERAD Tests „Wortliste“ und „Tiere benennen“
- Summenscore der Geriatrischen Depressionsskala (GDS)
- Mobilität gemessen durch den Timed Up & Go – Test
- Gesundheitsbezogene Lebensqualität (EQ-5D-5L)
- Aktivitäten des täglichen Lebens gemessen mittels des Barthel-Index
- Gewicht

Die statistischen Analysen umfassen detaillierte deskriptive Statistiken (Häufigkeiten, Lage- und Streuparameter) für die Parameter (gesamt und getrennt nach Interventionsgruppe) zur Beschreibung der Studienpopulation (Alter, Geschlecht, Bildung nach CASMIN, Frailty-

Index, etc.) sowie zur Beschreibung des primären Outcomes und der sekundären Endpunkte.

Für die Analyse des primären Zielkriteriums wird ein gemischtes Regressionsmodell angewandt. Als Random-Effekt wird die Praxis- bzw. Arztzugehörigkeit gekennzeichnet durch die PraxisID oder ArztID in das Modell eingeschlossen, als feste Effekte gehen neben der Gruppe (Kontrolle oder Intervention) die Faktoren Alter, Geschlecht und Ko-Morbiditäten zur Adjustierung ein. Vorgesehen ist ein lineares gemischtes Modell mit der Zielvariablen „Anzahl Krankenhausaufenthalte", entsprechend der tatsächlichen Verteilung der Zielvariablen kann auch ein ordinales logistisches oder ein Poisson Modell angewandt werden.

Die sekundären Endpunkte werden abhängig von der Verteilung mittels gemischter linearer Regressionsmodelle oder gemischter logistischer Regressionsmodell (binär oder ordinal) analysiert. Dabei wird Die Praxiszugehörigkeit als zufälliger Effekt in das Modell eingeschlossen, feste Effekte sind die Gruppenzughörigkeit, Alter, Geschlecht sowie Komorbiditäten.

Als Alternative zu den klassischen Regressionsverfahren werden die Methoden der Classification Tree Analyse (CART) angewandt. Bei diesem Verfahren wird nach Untergruppen von Fällen gesucht, die hinsichtlich der Verteilung der Response möglichst homogen sind. Die Charakterisierung von homogenen Untergruppen erfolgt dabei mit Hilfe eines binären Baumes: zunächst wird das gesamte Sample in zwei Teilräume aufgespaltet. Jeder so entstandene Teilraum und alle weiteren Teilräume können sukzessive weiter aufgesplittet werden, so dass insgesamt eine hierarchisch strukturierte Aufteilung entsteht. Die CART-Analyse wird mit 10-facher Kreuzvalidierung durchgeführt. Die Cut-Points der Prädiktoren werden durch das Verfahren bestimmt; fehlende Werte in den Prädiktoren werden durch surrogate Variablen ersetzt. Zielparameter sind in den jeweiligen CART-Analysen die Endpunkte (primärer und sekundäre); Prädiktoren sind die Gruppenzugehörigkeit (Kontrolle oder Intervention), Alter, Geschlecht, Ko-Morbiditäten und weitere mögliche Einflussfaktoren. Vorteile dieser Verfahren sind die Verteilungsfreiheit der Prädiktoren sowie die einfach interpretierbare Ergebnisdarstellung als Entscheidungsregel.

Die Auswertung der Safety-Parameter (insbesondere Mortalität und Hospitalisierungen) werden in regelmäßigen Abständen deskriptiv als Häufigkeiten verblindet (d.h. es ist nicht erkennbar, welche Gruppe die Intervention und welche die Kontrolle darstellt) dem Data Safety Monitoring Board (DSMB) zur Verfügung gestellt.

4. Ziel, Endpunkte und statistische Analysen der gesundheitsökonomischen Evaluation

Das Ziel der gesundheitsökonomischen Evaluation ist es die Effizienz der Intervention zu ermitteln indem Kosten und Outcomes der Interventionsgruppe mit den Kosten und Outcomes der Kontrollgruppe (care as usual) verglichen werden. Dabei werden alle Kosten im Zusammenhang mit der Intervention sowie mit der Inanspruchnahme von Gesundheitsleistungen (Krauth et al., 2005) aus Sicht der Sozialversicherung in Deutschland (Krankenversicherung, Pflegeversicherung und Rentenversicherung).

Um die Effizienz der Intervention zu bestimmen, wird eine Kosten-Effektivitätsanalyse und eine Kosten-Nutzwertanalyse durchgeführt. Errechnet wird das inkrementelle Kosten-Effektivitäts-Verhältnis (ICER: zusätzliche Kosten für jede zusätzlich vermiedene Krankenhauseinweisung) und das inkrementelle Kosten-Nutzwert-Verhältnis (ICUR:

zusätzliche Kosten für jedes zusätzlich gewonnene qualitätsadjustierte Lebensjahr (QALY)), als Quotient der Kosten- und der Nutzendifferenzen zwischen Interventionsgruppe und Kontrollgruppe. QALYs sind Nutzwerte und werden durch die Erfassung von gesundheitsbezogener Lebensqualität mit einem etablierten präferenzbasierten Lebensqualitätsinstrument auf Basis des EQ-5D-5L berechnet (Herdman et al., 2011) und mit einem deutschen Tarif (Greiner et al., 2005) bewertet um Nutzwerte zu generieren.

95% Konfidenzintervalle für den Outcome und die Kosten werden nicht-parametrisch aufgrund der Verteilungscharakteristika mittels Bootstrap-Prozeduren ermittelt (Briggs, 1997). Zur Berücksichtigung von Unsicherheit werden univariate and probabilistische Sensitivitätsanalysen durchgeführt und Kosten-Effektivitäts-Akzeptanzkurven angefertigt (Fenwick, 2004).

5. Analysepopulation

Die Auswertung der primären Zielgröße erfolgt nach dem *Intention to Treat* (ITT) Prinzip. In diesem Fall bedeutet dies, dass alle Patienten, die in die Studie eingeschlossen werden, d.h. alle vorgesehenen Patienten der Interventionsgruppe und alle Patienten der Kontrollgruppe, die ihre Studieneinwilligung geben, in die Analyse einbezogen werden. Dabei verbleiben die Patienten für die Analyse in der ursprünglichen Gruppe, auch wenn z.B. bei einem Patienten in der Interventionsgruppe die Familienkonferenzen nicht durchgeführt werden.
Bei vorzeitigem Abbruch der Studie wird als primärer Endpunkt die Anzahl Hospitalisierungen bis zum Zeitpunkt des Abbruchs eingesetzt, d.h. es wird nach dem *Last observation carried forward* (LOCF) Prinzip vorgegangen.

Als Sensitivitätsanalyse wird eine *per protocol* (PP) Analyse durchgeführt, d.h. in diese Analyse werden alle Patienten einbezogen, die die Studie ordnungsgemäß durchlaufen haben.

6. Data Handling

Fehlende Werte werden *nicht* a priori ersetzt. Einige der oben genannten Verfahren (CART, RPA) können mit fehlenden Werten umgehen, d.h. fehlende Werte werden durch sog. Surrogatvariablen nur bei den Split-Kriterien ersetzt. Um mögliche Verzerrungen durch fehlende Werte, die nicht oder nicht vollständig zufällig fehlen, zu untersuchen, werden Sensitivitätsanalysen durchgeführt.

7. Verwendete Software

Die Auswertungen werden mit den Softwarepaketen SPSS, STATA, SAS, und CART durchgeführt.

8. Referenzen

Breiman L, Friedman JH, Olshen RA, Stone CJ. Classification and Regression Trees. Chapman & Hall (Wadsworth, Inc.): New York, 1984.

Briggs, A.H., D.E. Wonderling, and C.Z. Mooney, Pulling cost-effectiveness analysis up by its bootstraps: a non-parametric approach to confidence interval estimation. Health economics, 1997. 6(4): p. 327–340.

Fenwick, E., B.J. O'Brien, and A. Briggs, Cost-effectiveness acceptability curves--facts, fallacies and frequently asked questions. Health economics, 2004. 13(5): p. 405–415.

Greiner W, Claes C, Busschbach JJ, von der Schulenburg JM. Validating the EQ-5D with time trade off for the German population. The European journal of health economics : HEPAC : health economics in prevention and care. Jun 2005;6(2):124-130.

Herdman, M., et al., Development and preliminary testing of the new five-level version of EQ-5D (EQ-5D-5L). Qual Life Res, 2011. 20(10): p. 1727-36.

Krauth, C. et al. (2005). Empirical standard costs for health economic evaluation in Germany- a proposal by the working group methods in health economic evaluation. Gesundheitswesen, 67(10), 736-746.
