# Supplementary material for: Family Conferences to Facilitate Deprescribing in Older Outpatients With Frailty and With Polypharmacy: The COFRAIL Cluster Randomized Trial
Source: JAMA Netw Open. 2023 Mar 27;6(3):e234723. doi: 10.1001/jamanetworkopen.2023.4723 (PMC10043750; doi:10.1001/jamanetworkopen.2023.4723)
Supplement: Supplement 2. — eTable. Geriatric Assessments and Safety Parameters at Baseline (T0) and After 12 Months (T2) eReferences [file jamanetwopen-e234723-s002.pdf]

Supplementary Online Content

Mortsiefer A, Löscher S, Pashutina Y, et al. Family conferences to facilitate deprescribing in older outpatients with frailty and with polypharmacy: the COFRAIL pragmatic cluster randomized trial. *JAMA Netw Open*. 2023;6(3):e234723. doi:10.1001/jamanetworkopen.2023.4723

eTable. Geriatric Assessments and Safety Parameters at Baseline (T0) and After 12 Months (T2)

eReferences

This supplementary material has been provided by the authors to give readers additional information about their work.

© 2023 Mortsiefer A et al. *JAMA Network Open.*

eTable. Geriatric assessments and safety parameters at baseline (T0) and after 12 months (T2)

Geriatric assessments						
	**No. (%)**					
**Variable**	**Intervention group**		**Control group**			***P* value**
	T0	T2	T0	T2		
**Cognition[1]**					**Mixed Linear Regression [a] β [95% CI]**	
CERAD Animals, mean number of correct mentions within 60 sec. (SD)	14.10 (5.58) (N=192)	14.40 (6.17) (N=171)	14.06 (5.48) (N=178)	14.46 (5.44) (N=152)	0.32 [-0.58 -1.22]	.49
CERAD Word List Memory, mean total of 3 runs (SD)	15.18 (6.04) (N=180)	17.60 (6.73) (N=149)	15.04 (5.50) (N=168)	16.94 (6.13) (N=132)	0.97 [-0.33 - 2.27]	.15
CERAD Word List Recall, mean (SD)	4.69 (2.34) (N=169)	5.94 (2.91) (N=144)	4.73 (2.36) (N=160)	5.47 (2.70) (N=127)	0.53 [-0.76 - 1.13]	.087
**Geriatric Depression Scale (GDS)[2] [b]**					**Mixed Ordinal Logistic Regression [a] OR [95% CI]**	
normal	154 (81.9)	129 (78.2)	136 (76.4)	112 (75.2)	0.74 [0.33 - 1.71]	.49
light to moderate	24 (12.8)	27 (16.4)	37 (20.8)	31 (20.8)		
severe	10 (5.3)	9 (5.5)	5 (2.8)	6 (4.0)		
**Barthel Index[3]** (functional restriction)					**Mixed Ordinal Logistic Regression [a] OR [95% CI]**	
U50.00 Not or low	37 (18.5)	32 (16.1)	26 (14.1)	29 (15.8)	1.21 [0.78 - 1.87]	.39
U50.10 Slight	95 (47.5)	87 (43.7)	83 (44.9)	82 (44.6)		
U50.20 Medium	50 (25.0)	53 (26.6)	51 (27.6)	42 (22.8)		
U50.30 Moderate	11 (5.5)	15 (7.5)	13 (7.0)	19 (10.3)		
U50.40 Severe	7 (3.5)	10 (5.0)	8 (4.3)	7 (3.8)		
U50.50 Very severe	0 (0.0)	2 (1.0)	4 (2.2)	5 (2.7)		
**Body weight [d]**					**Mixed Ordinal Logistic Regression [a] OR [95% CI]**	
Underweight	5 (2.7)	2 (1.1)	1 (0.6)	1 (0.6)	1.317 [0.78 - 2.24]	.31
Normal weight	55 (29.6)	55 (30.9)	39 (23.8)	43 (26.9)		
Overweight	64 (34.4)	67 (37.6)	56 (34.1)	49 (30.6)		
Obesity gr. I	39 (21.0)	30 (16.9)	43 (26.2)	44 (27.5)		
Obesity gr. II	16 (8.6)	14 (7.9)	16 (9.8)	15 (9.4)		
Obesity gr. III	7 (3.8)	10 (5.6)	9 (5.5)	8 (5.0)		
**Number of falls [d]** (last 6 months)					**Mixed Ordinal Logistic Regression [a] OR [95% CI]**	
None	123 (63.4)	134 (67.7)	123 (66.5)	132 (73.3)	1.16 [0.72 -1.87]	.54
1-2	46 (23.7)	54 (27.3)	49 (26.5)	41 (22.8)		
3-5	19 (9.8)	8 (4.0)	10 (5.4)	6 (3.3)		
6-10	4 (2.1)	1 (0.5%	2 (1.1)	0 (0.0)		
> 10	2 (1.0)	1 (0.5)	1 (0.5)	1 (0.6)		

© 2023 Mortsiefer A et al. *JAMA Network Open.*

**Use of emergency services** [d] (at least 1x in the last 6 months)					**Mixed Binary Logistic Regression** [a] **OR (95% CI)**	***P* value**
Yes	31 (11.4)	19 (9.2)	24 (9.8)	21 (11.4)	0.75 [0.34 - 1.63] (P = .46)	.46
No	240 (88.6)	188 (90.8)	222 (90.2)	163 (88.6)		
**Safety parameters**						
**Variable**	**Intervention group**		**Control group**		**Mixed Linear Regression** [a] **β [95% CI]**	***P* value**
	T0	T2	T0	T2		
**Blood pressure** mmHg [c]	N=260	N=197	N=252	N=188		
Systolic, mean (SD)	131.32 (17.85)	130.81 (15.83)	132.98 (16.28)	134.63 (18.08)	-2.31 [-5.93 - 1.31]	.21
Diastolic, mean (SD)	75.52 (9.75)	77.13 (9.48)	75.64 (9.21)	76.88 (9.18)	0.71 [-1.50 - 2.93]	.53
**Heart rate** per min [c]	N=236	N=158	N=239	N=163		
Mean value (SD)	72.92 (9.70)	73.59 (10.51)	71.86 (9.72)	73.70 (11.3)	-0.40 [-3.08 - 2.27]	.77
**Fasting glucose** mg/dl [c]	N=244	N=140	N=227	N=145		
Mean value (SD)	85.41 (59.48)	91.24 (60.64)	78.74 (56.08)	84.26 (63.94)	1.76 [-7.75 - 11.28]	.72
**GFR** [c]	N=253	N=170	N=238	N=163		
Mean value (SD)	55.04 (19.62)	52.32 (19.31)	53.87 (19.17)	53.11 (19.57)	-0.08 [-2.46 - 2.31]	.95

[a] Each adjusted for age, gender and baseline results
[b] The maximum score of the GDS-15 is 15 points with the following classification: 0-5 normal, 6-10 mild to moderate depression, 11-15 severe depression
[c] Adjusted for age, sex, and baseline results
[d] Self-reported by the participants

As the data collection by the study nurses had to be changed from home visits to telephone interviews in the course of the COVID-19 pandemic, the grip strength measurement and the "Timed Up & Go" test could not be performed in relevant numbers at T2.

For all results of these parameters, there were no statistically significant differences between the intervention and the control group.

OR = odds ratio; β = regression coefficient; SD = standard deviation; 95% CI = 95% confidence interval; GFR = glomerular filtration rate

© 2023 Mortsiefer A et al. *JAMA Network Open.*

eReferences

1. Stein J, Luppa M, Luck T, et al. The assessment of changes in cognitive functioning: age-, education-, and gender-specific reliable change indices for older adults tested on the CERAD-NP battery: results of the German Study on Ageing, Cognition, and Dementia in Primary Care Patients (AgeCoDe). Am J Geriatr Psychiatry. 2012;20(1):84-97. Medline:22183013 doi:10.1097/JGP.0b013e318209dd08

2. Yesavage JA, Brink TL, Rose TL, et al. Development and validation of a geriatric depression screening scale: a preliminary report. J Psychiatr Res. 1982-1983;17(1):37-49. Medline:7183759 doi:10.1016/00223956(82)90033-4 17

3. Wade DT, Collin C. The Barthel ADL Index: a standard measure of physical disability? Int Disabil Stud. 1988;10(2):64-67. Medline:3042746 doi:10.3109/09638288809164105

© 2023 Mortsiefer A et al. *JAMA Network Open.*
