# Supplementary material for: Family Conferences to Facilitate Deprescribing in Older Outpatients With Frailty and With Polypharmacy: The COFRAIL Cluster Randomized Trial
Source: JAMA Netw Open. 2023 Mar 27;6(3):e234723. doi: 10.1001/jamanetworkopen.2023.4723 (PMC10043750; doi:10.1001/jamanetworkopen.2023.4723)
Supplement: Supplement 3. — Data Sharing Statement [file jamanetwopen-e234723-s003.pdf]

Data Sharing Statement

Mortsiefer. Family Conferences to Facilitate Deprescribing in Older Outpatients With Frailty and With Polypharmacy. *JAMA Netw Open*. Published March 27, 2023. doi:10.1001/jamanetworkopen.2023.4723

Data
Data available: No

Additional Information
Explanation for why data not available: Data cannot be made available, owing to the European Union General Data Protection Regulation. Readers who are interested in study materials (e.g., model consent form) should contact the corresponding author.
